# Identification of Nanog as a novel inhibitor of Rad51

**DOI:** 10.1038/s41419-022-04644-9

**Published:** 2022-02-26

**Authors:** Ying Xin, Juanjuan Wang, Yahong Wu, Qianqian Li, Mingyang Dong, Chang Liu, Qijia He, Ruifeng Wang, Dian Wang, Sen Jiang, Wei Xiao, Yang Tian, Weiwei Zhang

**Affiliations:** 1 College of Life Sciences, Capital Normal University, Beijing, China; 2 Department of Chemistry, Capital Normal University, Beijing, China

**Keywords:** Double-strand DNA breaks, Target identification

## Abstract

To develop inhibitors targeting DNA damage repair pathways is important to improve the effectiveness of chemo- and radiotherapy for cancer patients. Rad51 mediates homologous recombination (HR) repair of DNA damages. It is widely overexpressed in human cancers and overwhelms chemo- and radiotherapy-generated DNA damages through enhancing HR repair signaling, preventing damage-caused cancer cell death. Therefore, to identify inhibitors of Rad51 is important to achieve effective treatment of cancers. Transcription factor Nanog is a core regulator of embryonic stem (ES) cells for its indispensable role in stemness maintenance. In this study, we identified Nanog as a novel inhibitor of Rad51. It interacts with Rad51 and inhibits Rad51-mediated HR repair of DNA damage through its C/CD2 domain. Moreover, Rad51 inhibition can be achieved by nanoscale material- or cell-penetrating peptide (CPP)-mediated direct delivery of Nanog-C/CD2 peptides into somatic cancer cells. Furthermore, we revealed that Nanog suppresses the binding of Rad51 to single-stranded DNAs to stall the HR repair signaling. This study provides explanation for the high γH2AX level in unperturbed ES cells and early embryos, and suggests Nanog-C/CD2 as a promising drug candidate applied to Rad51-related basic research and therapeutic application studies.

## Introduction

Endogenous metabolites and environmental agents, such as radiation and chemical mutagens, can result in DNA damages in eukaryotic cells [1–3]. To avoid of DNA damage accumulation and maintain genome integrity, cells employ sophisticated repair pathways mainly including base excision repair, nucleotide excision repair, mismatch repair, non-homologous end-joining (NHEJ) pathway and homologous recombination (HR) repair [4, 5]. The classical NHEJ (C-NHEJ) and HR repair pathway are two principal mechanisms to repair double-strand breaks (DSBs) that are most dangerous DNA damage type [6–9]. DSBs are two adjacent single-stranded nicks within 20-base pair distance coexisting in both DNA strands [10]. Failure in DSB repair results in severe cellular consequences, including gene mutations, chromosome aberrations and even cell death [11]. To repair DSBs, the C-NHEJ pathway directly ligates two blunt ends after removal of damaged nucleotides, whereas HR-mediated repair signaling requires a chromatid containing an intact sequence homologous to the lesion DNA [12, 13]. Therefore, C-NHEJ is fast but mutagenic while HR is typically error-free [14]. Moreover, initiation of the HR pathway requires DNA resection during which the broken DNA ends are resected to produce single-stranded DNA (ssDNA) overhangs at 3’ end, whereas NHEJ is independent of this step [14, 15]. Although both C-NHEJ and HR can faithfully repair DSBs, cells make a choice from these two pathways in a cell cycle-dependent manner [12, 13, 16–18]. C-NHEJ-mediated DSB recovery can ubiquitously occur in all cell cycle phases but predominantly acts in the G0/G1 and G2 phases. HR is specifically restricted to post-replicative S and G2 phase, and its highest activity occurs in S phase [14, 17]. At the molecular level, choice of these two repair pathways can be largely determined by several critical proteins. For instance, Rad51 recombinase directly binds to the overhanging single-stranded DNA tails to activate HR, while p53-binding protein 1 (53BP1) binding of DNA lesions blocks DNA end resection, which consequently prevents HR but promotes C-NHEJ [19–22].

Embryonic stem (ES) cells are derived from the inner cell mass (ICM) of early embryos [23, 24]. Similar with the in vivo ICM cells, ES cells possess pluripotency to differentiate into all types of somatic cells in organisms, which determines their application potential in the field of degenerative disease therapy [25]. Interestingly, ES cells exhibit much lower mutation rate than somatic cells under equal damage challenges [26–29], indicating that ES cells prefer error-free repair pathways to efficiently erase DNA lesions so as to avoid of damage transmission into progeny cells. In the presence of DSBs, ES cells predominantly employ S phase-favored error-free HR repair pathway, rather than G1/G2-favored mutagenic C-NHEJ, which is possibly due to their unique cell cycle structure with highly-accumulated S phase but shortened G1 and G2 phases [14, 30–33]. Consistently, ES cells maintain higher expressions of HR-related factors, including Rad51, Rad52 and Rad54, than somatic cells. Moreover, upon ES cell differentiation, the HR repair signaling activity is reduced but C-NHEJ is induced [29, 34]. Intriguingly, ES cells exhibit constitutive activation of phosphorylated H2AX at serine 139 (γH2AX) in absence of any exogenous genotoxic agent [31, 35–37]. γH2AX typically acts as one of the earliest markers specifically responding to DSBs, and is always employed to assess the efficiency of DSB repair [38–40]. Noteworthily, γH2AX accumulation in ES cells seems not due to any artificial effect in culture in vitro since it is similarly observed throughout the unperturbed preimplantation embryos in vivo including the ICM [37, 41]. Moreover, positive foci of Rad51, rather than 53BP1, are detected in ES cells without DNA damage induction [41, 42]. Thus, some ES cell-specific regulators might impede the activity of Rad51 to sustain γH2AX accumulation for stemness maintenance.

Interestingly, using proteomic approaches, Gagliardi et al. identifies Nanog as a putative interaction partner of Rad51 [43]. Nanog is a core transcription factor of ES cells that cooperates with Octamer-binding transcription factor 4 (Oct4) and Sex determining region Y-box 2 (Sox2) to largely determine stemness-specific gene expression profile [44, 45]. In this scenario, we speculated that Nanog could act as an ES cell-specific inhibitor of Rad51. In this study, we demonstrated that Nanog is capable of directly interacting with Rad51 to repress Rad51-promoted DNA damage repair and consequently maintains the high basal level of γH2AX in ES cells. Importantly, Nanog-mediated Rad51 inhibition is not restricted to ES cells. Vector-mediated overexpression or direct protein delivery of either the full-length or C terminal domains of Nanog into somatic cancer cells achieved strong inhibitory effect on Rad51 activity. Furthermore, we revealed that Nanog impedes the binding of Rad51 to ssDNAs and reduces HR efficiency. The findings extend our understanding of ES cell biology, and provide Nanog fragments as putative inhibitor drugs for Rad51-associated basic research and therapeutic application studies in future.

## Results

### Nanog interacts with Rad51 and promotes γH2AX accumulation

**Fig. 1:**
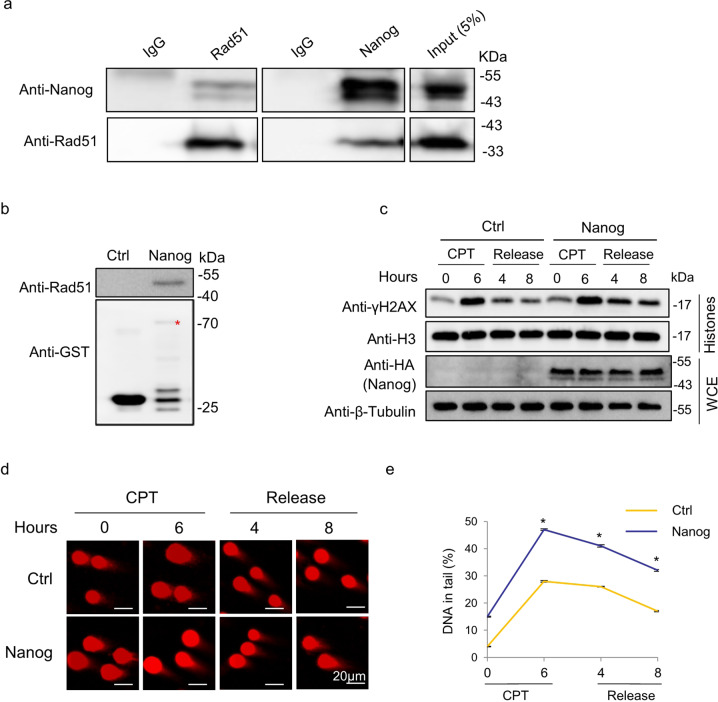
Nanog interacts with Rad51 and promotes γH2AX accumulation in mouse ES cells. **a** Co-IP assay was used to detect the association of Nanog with Rad51. Mouse ES cell extracts were subjected to co-IP with the antibody against Nanog or Rad51, followed by western blotting with the antibodies against Rad51 and Nanog, respectively. Whole cell extract for IP was used as the input control. **b** Pull-down assay was performed to analyze the direct association of Nanog with Rad51. Bacterially purified GST-tagged Nanog was conjugated to glutathione-sepharose beads, and subsequently incubated with purified His-tagged Rad51. The elution was analyzed by western blot using the anti-Rad51 antibody. GST was used as a control. **c** Nanog overexpression retarded γH2AX removal. Mouse ES cells expressing HA-tagged Nanog were treated with CPT for 6 h. The medium was changed to fresh CPT-free medium and allowed cells to grow for additional 4 and 8 h, respectively. Whole cell proteins and histones were extracted and subjected to SDS-PAGE. The cells transfected with the mock vector were used as controls. **d** Representative cell images of the comet assay. Similar CPT treatment and release strategies with **c** were performed for the Nanog-overexpressed mouse ES cells and mock control cells. **e** The average percentages of DNA in tails were calculated. The data are based on three independent repeats, and presented as mean ± SEM. *p < 0.05 (Student’s *t*-test).

To investigate the association between Nanog and Rad51, first of all, we performed a co-immunoprecipitation (co-IP) assay with the whole cell extracts of mouse ES cells and confirmed that these two factors form a complex in vivo (Fig. 1a). Furthermore, the pull-down assay showed that bacterially purified Nanog and Rad51 proteins can physically interact with each other (Fig. 1b). The robust affinity of Nanog with Rad51 implicates its potential involvement in regulating Rad51-mediated HR repair signaling. Since γH2AX monitors the effect of Rad51-mediated DNA damage repair [39], we checked whether Nanog is implicated in the process of γH2AX removal. We treated ES cells with camptothecin (CPT) for 6 h and subsequently grew them in the CPT-free medium for additional 4 and 8 h, respectively, to allow cell recovery from CPT-induced DSBs. Compared with the vector-transfected control cells where γH2AX was gradually repaired after CPT removal, *Nanog* overexpression dramatically retarded γH2AX removal (Fig. 1c). Consistently, the comet assay under neutral condition in ES cells showed that Nanog overexpression increased percentage of DNA in tails (Fig. 1d, e). Moreover, after CPT removal, although the extensively spread comet tails of the control cells were efficiently repaired, *Nanog*-elevated cells sustained higher extent of tail DNA (Fig. 1d, e). Of note, overexpression of the other two core stemness factors, Oct4 and Sox2, failed in activating γH2AX (Supplementary Fig. S1a, b). Consistently, neither of these two factors interacts with Rad51 in ES cells (Supplementary Fig. S1c).

**Fig. 2:**
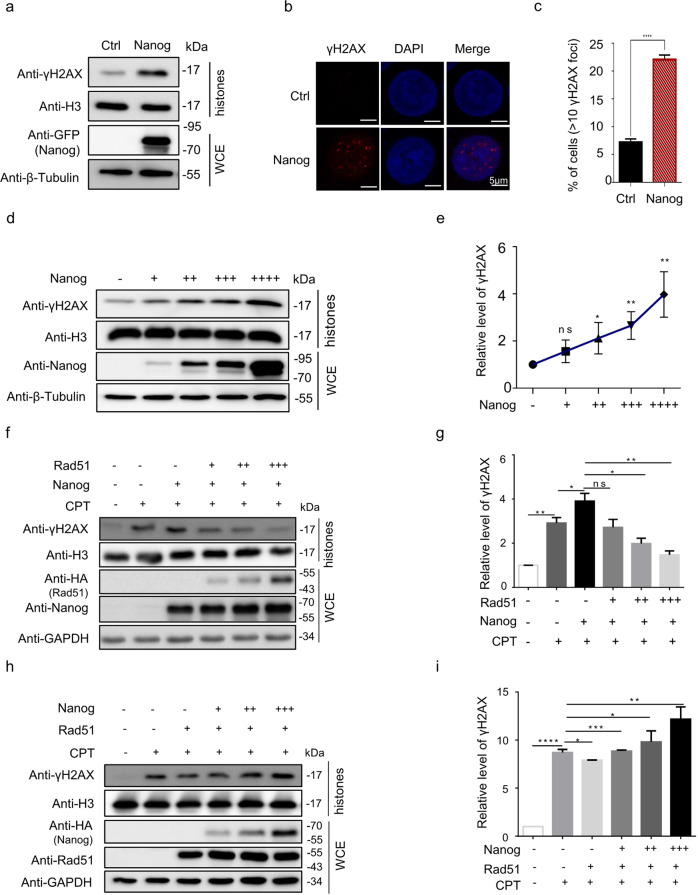
Nanog inhibits Rad51-mediated γH2AX removal in human cancer cells. **a** Nanog enhanced level of γH2AX. The plasmid expressing GFP-tagged Nanog was transfected in HeLa cells for 48 h. Mock vector was used as control. Whole cell proteins and histones were extracted and subjected to SDS-PAGE, respectively. **b**, **c** Nanog overexpression increased γH2AX foci. HeLa cells overexpressing GFP-tagged Nanog were stained with the anti-γH2AX antibody (red) and DAPI (blue). The nuclei containing more than 10 foci were considered to be positive. **d** Nanog-promoted γH2AX activation. 293 cells were transfected with different amounts of plasmid expressing GFP-tagged Nanog (+: 2 μg; ++: 4 μg; +++: 6 μg; ++++: 8 μg). Whole cell proteins and histones were extracted and subjected to SDS-PAGE, respectively. **e** The blot band intensities from **d** were quantitated by MultiGauge software (Fujifilm). The data was normalized to loading controls with the antibody against H3. **f** HeLa cells ectopically expressing Nanog were transfected with different amounts of plasmid expressing HA-tagged Rad51 (+: 0.1 μg; ++: 0.5 μg; +++: 1 μg). Whole cell proteins and histones were extracted and subjected to SDS-PAGE, respectively. **g** The blot band intensities of **f** were quantitated by MultiGauge software (Fujifilm). The data was normalized to loading controls with the antibody against H3. **h**, **i** HeLa cells ectopically expressing Rad51 were transfected with different amounts of plasmid expressing HA-tagged Nanog (+: 1 μg; ++: 2 μg; +++: 4 μg). Similar strategies were performed with **f** and **g**. The data are based on three independent repeats, and presented as mean ± SEM. ***p < 0.001; **p < 0.01; *p < 0.05 (Student’s *t*-test).

It is known that to develop novel inhibitors targeting Rad51 acts as an important avenue to improve the effectiveness of chemo- or radiotherapy for cancer patients. Therefore, we sought to examine whether Nanog could inhibit endogenous Rad51 in human cancer cells. However, high expression of Nanog is restricted to pluripotent cells rather than somatic cells [44]. Although Nanog displays abnormally activated in some human cancers, such as breast, ovarian, liver and colorectal cancers, its expression is modest in general [46–49]. In this scenario, we investigated whether the Nanog fragment derived from mouse ES cells could serve as an exogenous peptide inhibitor to interrupt endogenous Rad51 of human cancer cells. We overexpressed *Nanog* in HeLa cells. The western blotting assay with histone extracts revealed that Nanog overexpression markedly enhanced the overall level of γH2AX in absence of DNA damage agents (Fig. 2a). Moreover, Nanog elevation increased the number of γH2AX foci in chromatin (Fig. 2b, c). Through titrating amounts of the *Nanog-*expressing plasmid for transfection, we found that the levels of γH2AX were well correlated with the extent of Nanog elevation, suggesting that γH2AX accumulation specifically attributed to Nanog elevation (Fig. 2d, e). Moreover, consistent with the observation in mouse ES cells, the overexpressed exogenous Nanog in human HeLa cells sustained higher extent of tail DNA after CPT removal (Supplementary Fig. S2). To further investigate the inhibitory effect of Nanog on Rad51 in human cancer cells, we manipulated Rad51 expression in HeLa cells and found that gradual increase in Rad51 was well correlated with the extent of decrease in Nanog-activated γH2AX (Fig. 2f–g). On the other hand, gradual increase in Nanog resulted in corresponding enhanced inhibition of Rad51-mediated γH2AX removal (Fig. 2h, i).

Collectively, we conclude that Nanog acts as an effective inhibitor of Rad51 both in mouse ES cells and human cancer cells.

### The C and CD2 of Nanog can interact with Rad51 and activate γH2AX

**Fig. 3:**
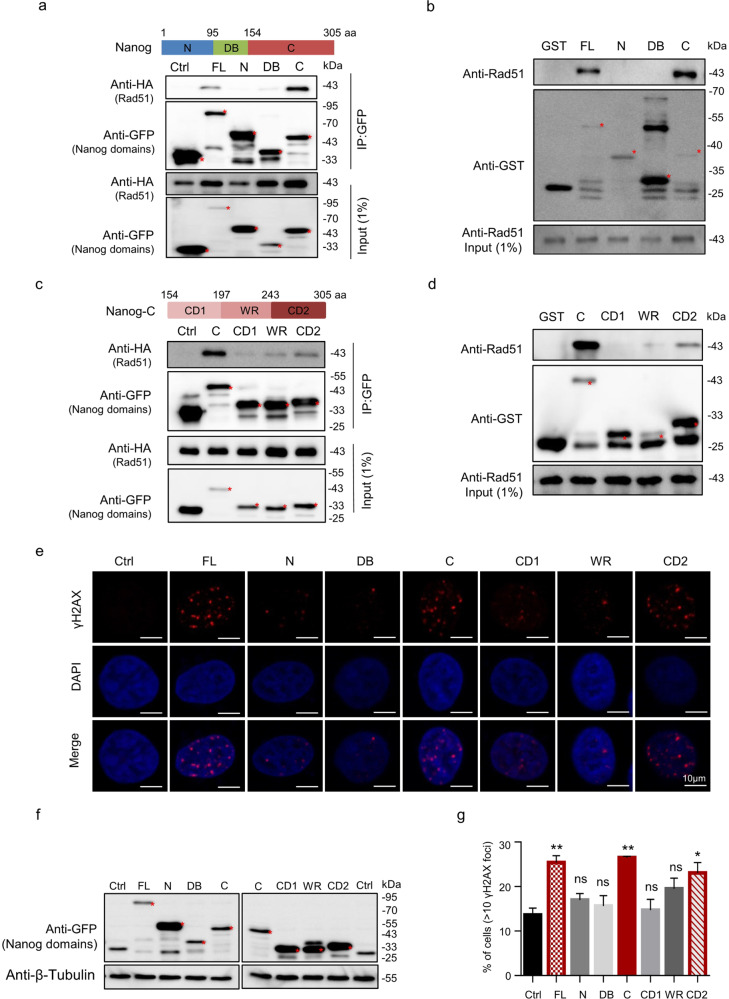
Identification of Nanog subregions interacting with Rad51. **a** Co-IP assay identified the regions of Nanog interacting with Rad51. Plasmids expressing HA-tagged Rad51 and GFP-tagged Nanog fragments were co-transfected into 293 cells. Cell extracts were subjected to GFP glutathione-sepharose beads, followed by western blotting with antibodies against HA and GFP. The upper panel: schematic diagram of Nanog domains. FL full-length Nanog, N N terminus of Nanog, DB DNA-binding homeodomain, C C terminus of Nanog. **b** Purified GST-tagged Nanog fragments were conjugated to Glutathione-sepharose beads to capture His-tagged Rad51. **c** Co-IP assay identified the subregions of C interacting with Rad51. Plasmids expressing HA-tagged Rad51 and GFP-tagged C fragments were co-transfected into 293 cells. Cell extracts were subjected to GFP glutathione-sepharose beads, followed by western blotting with antibodies against HA and GFP. The upper panel: schematic diagram of the subregions in the C terminus. CD1 C terminal domain 1, WR tryptophan repeat domain, CD2 C terminal domain 2. **d** Purified GST-tagged Nanog-C fragments were conjugated to Glutathione-sepharose beads to capture His-tagged Rad51. **e** C and CD2 activate γH2AX foci. HeLa cells overexpressing GFP-tagged Nanog fragments were stained with the anti-γH2AX antibody (red) and DAPI (blue). **f** Western blotting assay examined the expression levels of Nanog fragments. **g** γH2AX foci in the cells from **e** were counted and analyzed. The nuclei containing more than 10 foci were considered to be positive. The data are based on three independent repeats, and presented as mean ± SEM. ***p < 0.001; **p < 0.01; *p < 0.05 (Student’s *t*-test).

Nanog contains well-characterized domains including the serine-rich N terminus, DNA-binding homeodomain (DB), and C terminus. The C terminus harbors the C terminal domain 1 (CD1) and CD2 separating by a tryptophan repeat (WR) domain [50]. To further characterize the effect of Nanog on repressing Rad51 activity, we sought to identify the critical subregion(s) of Nanog mediating its association with Rad51. To this end, we constructed a series of Nanog truncations specifically expressing the N, DB and C domains, respectively. Both the in vivo co-IP assay and in vitro pulldown experiment results showed that the C terminus, rather than the N or DB, is able to interact with Rad51 (Fig. 3a, b). Interestingly, all three subregions of the C terminus were capable of associating with Rad51. However, the CD2 exhibited the highest affinity with Rad51, compared with CD1 and WR (Fig. 3c, d).

Next, we sought to determine the capability of individual Nanog domains in regulating γH2AX accumulation. The immunocytochemistry (ICC) staining analysis revealed that the C and CD2 both displayed similar capability with full-length Nanog in promoting γH2AX foci accumulation (Fig. 3e–g). The N terminus and DB failed in any induction. Although the CD1 and WR exhibited weak affinity in associating Rad51, neither of their overexpression enhanced the number of γH2AX foci (Fig. 3e–g). Moreover, western blotting assays revealed that both the C and CD2, rather than the N, were able to increase the overall level of γH2AX in absence of any damage-inducing agent in human 293 cells (Supplementary Fig. S3a–c). Consistently, the comet assay under neutral condition revealed that overexpressing either C or CD2 in 293 cells increased percentages of tail DNA (Supplementary Fig. S3d, e). Moreover, elevated expression of each fragment could further aggravate CPT-induced DSBs and markedly retard damage repair after CPT removal (Supplementary Fig. S3d, e). Of note, although full-length Nanog elevation slightly decreased the number of cells in G2/M phase, this could not act as the dominant cause for Nanog-promoted γH2AX accumulation since no obvious cell cycle change was detected after overexpression of either Nanog-C or CD2 (Supplementary Fig. S4).

### Nanog inhibits the activity of Rad51 in the HR repair signaling

**Fig. 4:**
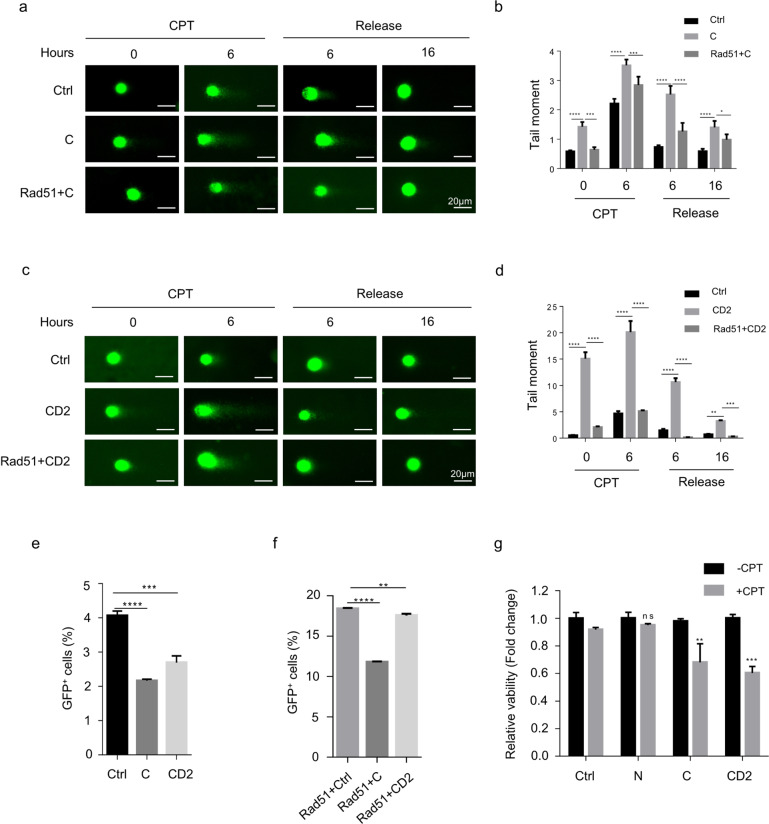
Nanog-C and CD2 inhibits the activity of Rad51. **a** Representative cell images of the comet assay. HeLa cells co-expressing Nanog-C and Rad51 were treated with CPT for 6 h. The cells transfected with mock vectors and the one expressing Nanog-C, respectively, were used as controls. The medium was changed to fresh CPT-free medium and allow cells to grow for additional 6 and 16 h, respectively. **b** Tail moments from **a** were calculated. **c**, **d** Similar experiments and analysis with **a** and **b** were performed to examine the rescue effect of Rad51 on CD2-prevented DSB repair. **e** HR reporter assay showing the effect of Nanog-C and CD2 on HR-mediated DNA damage repair. HR-Flex cell lines were transfected with the plasmids expressing I-SceI and Nanog fragments, respectively. After 48 h, the number of GFP-positive cells was analyzed by flow cytometry. **f** HR reporter assay showing C- and CD2-mediated inhibition of Rad51 activity in the HR repair signaling. HR-Flex cell lines were co-transfected with the plasmids expressing I-SceI, Nanog fragments and Rad51, respectively. Similar analysis was performed with **e**. **g** MTT assay assessed the cell viability under CPT treatment. 293 cells expressing GFP-tagged Nanog fragments were analysed 12 h after treatment with 1 μM CPT. The data are based on three independent repeats, and presented as mean ± SEM. ****p < 0.0001; ***p < 0.001; **p < 0.01; *p < 0.05 (Student’s *t*-test).

Next, we investigated whether Nanog fragments-caused γH2AX accumulation attributes to Rad51 inhibition. Two constructs respectively expressing Nanog fragments and Rad51 were co-transfected into HeLa cells. The comet assay under neutral condition detected increased tailed DNA in the C-overexpressed cells in absence of CPT treatment, which, however, was successfully rescued by Rad51 elevation (Fig. 4a, the left panel; b). Similar observations were obtained under CPT treatment (Fig. 4a, the 2nd panel; b). Moreover, Rad51 overexpression prevented C elevation-caused delay of damage repair after CPT removal (Fig. 4a, the right two panels; b). Rad51 exhibited similar rescue effect in the CD2-overexpressed cells (Fig. 4c, d). To further confirm the inhibitory effect of Nanog on Rad51-mediated HR repair, we employed a HR reporter cell line (HR-Flex) harboring two EGFP expressing cassettes, one of which is inserted with a 330bp-sized Flex1 fragment in the middle and acts as a HR substrate because it can be recognized and cleaved by exogenously expressed I-SceI for DSB formation. The other EGFP cassette serves as the donor sequences for HR. I-SceI cleavage-induced HR restores GFP fluorescence signal [51]. In the control line, the Flex1 sequence is replaced by a 330 bp luciferase-expressing fragment (HR-Luc) [51]. HR efficiency was determined as the percentage of EGFP-positive HR-Flex cells, normalized to HR-Luc. We found that overexpression of either the C terminus or CD2 significantly impeded EGFP-positive cell generation (Fig. 4e). More specifically, both fragments were capable of reducing the efficiency of Rad51-mediated HR repair (Fig. 4f). Since impaired repair of DSBs severely threatens cell survival [6], we inferred that repression of Rad51 activity by Nanog fragments might sensitize cells to genotoxic agents. To demonstrate it, we employed survival assay to check the cellular sensitivity to CPT treatment. The viability of 293 cells was assessed by MTT assay after 12 h of 1 μM CPT treatment. Compared with the control, overexpression of the C terminus and CD2, rather than the N terminus, markedly compromised the viability of cells in the presence of CPT (Fig. 4g).

**Fig. 5:**
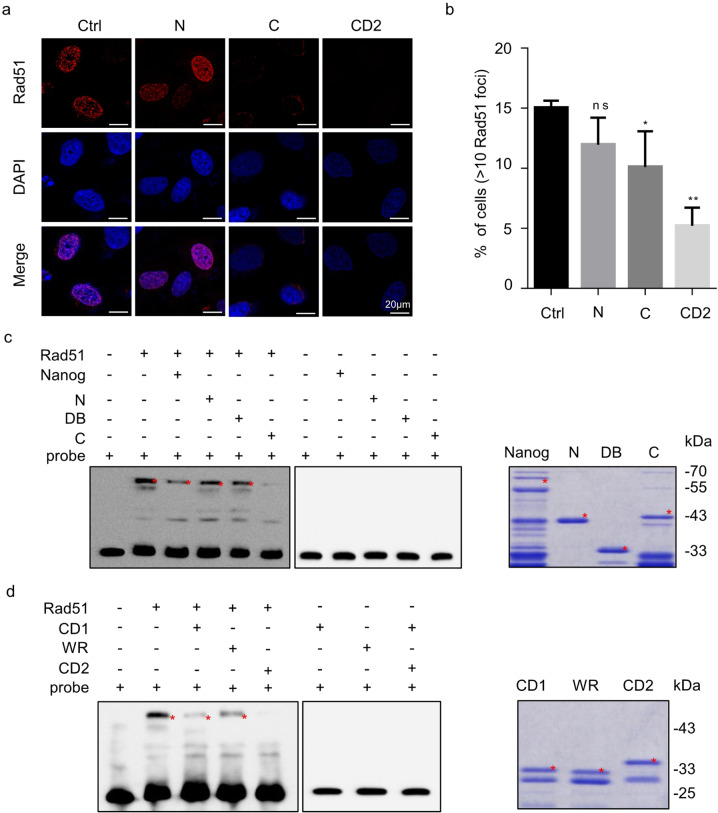
The mechanism underlying Nanog-mediated Rad51 inhibition. **a** HeLa cells overexpressing Nanog fragments were stained with the anti-Rad51 antibody (red) and DAPI (blue). **b** Rad51 foci in the cells from **a** were counted and analyzed. The nuclei containing more than 10 foci were considered to be positive. **c**, **d** EMSA assay showing the capability of full-length and fragments of Nanog in inhibiting Rad51 binding to ssDNA. The right panels show the results of the Coomassie brilliant blue staining of the purified Nanog fragments. The data are based on three independent repeats, and presented as mean ± SEM. **p < 0.01; *p < 0.05 (Student’s *t*-test).

Next, we sought to elucidate the mechanisms underlying Nanog-mediated Rad51 inhibition. We performed the ICC analysis in the cells co-expressing Nanog and Rad51 and found that full-length Nanog repressed CPT treatment-induced Rad51 foci formation (Supplementary Fig. S5a, b). Both the C terminus and CD2, rather than the N, displayed similar effect (Fig. 5a, b). By using the quantitative realtime PCR assay and western blotting assay, we found that neither the mRNA nor the protein level of Rad51 was changed by overexpression of Nanog fragments, which rules out the possibility that Nanog inhibits Rad51 foci at the lesion sites through altering its expression (Supplementary Fig. S5c, d). On the other hand, we observed increased 53BP1 foci in Nanog-overexpressed cells, independent of CPT treatment (Supplementary Fig. S6a). However, Nanog was not capable of interacting with 53BP1 (Supplementary Fig. S6b). Since 53BP1 promotes NHEJ through competing with Rad51 to repair DSBs, induction of 53BP1 foci could act as an indirect consequence after Nanog succeeds in inhibiting Rad51 accumulation at the lesion sites. In order to examine this speculation, we performed the pearson correlation coefficient analysis of the ICC staining results and found that upon CPT treatment, Nanog foci is highly correlated with Rad51, but not with 53BP1 foci (Supplementary Fig. S6c, d). These results demonstrate that the direct interplay occurs between Nanog and Rad51, but not 53BP1.

It is known that HR-mediated repair of DSBs requires Rad51 binding to the protruding ssDNA tail to form a helical nucleoprotein filament at the lesion loci [52–54]. Next, we asked whether Nanog interferes with the ssDNA binding capability of Rad51. By means of electrophoretic mobility shift assay (EMSA), we showed that the full-length Nanog and C terminus, rather than the N terminus and DB, displayed similar repressive effect on Rad51 binding of ssDNA (Fig. 5c). More specifically, the CD2 showed the strongest repressive effect compared with CD1 and WR (Fig. 5d). Collectively, we conclude that Nanog inhibits Rad51 through preventing its binding to ssDNA.

### Direct delivery of Nanog fragments into cells by nanoscale material or cell-penetrating peptide (CPP) achieves strong Rad51 inhibition

**Fig. 6:**
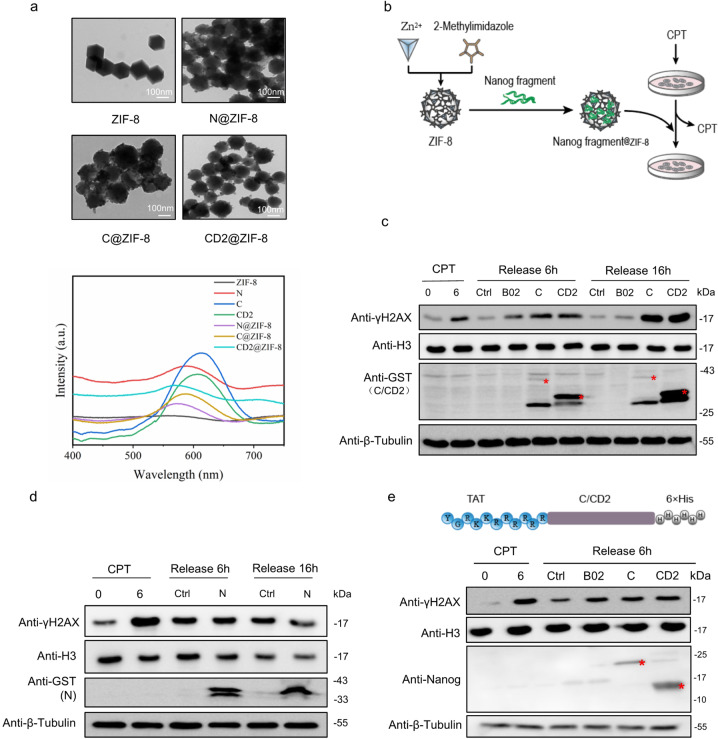
ZIF-8 or TAT tag-mediated direct delivery of Nanog fragments into cells achieves Rad51 inhibition. **a** TEM image (the upper panel) and UV-Vis spectra (the lower panel) of Nanog fragments@ZIF-8. **b** Schematic illustration of the experiment procedures. **c** C@ZIF-8 and CD2@ZIF-8 retarded γH2AX removal. HCT116 cells were treated with CPT for 6 h. The medium was changed to fresh CPT-free medium containing B02, C@ZIF-8 and CD2@ZIF-8, respectively, and allowed cells to grow for additional 6 and 16 h. Whole cell proteins and histones were extracted and subjected to SDS-PAGE. **d** N@ZIF-8 failed in delaying γH2AX removal. Similar experiments with **c** were employed. **e** TAT-mediated uptake of C and CD2 into cells retarded γH2AX removal. TAT-fused Nanog fragments were added into HCT116 after 6-h CPT treatment. The cells grew for additional 6 h. Whole cell proteins and histones were extracted and subjected to SDS-PAGE. The data are based on three independent repeats.

Rad51 is widely involved in regulating DNA damage repair, replication fork reversal and stabilization [55–63]. Its hyperactivity leads to radio- and chemotherapeutic resistance in cancer patients [64–71]. Therefore, the robust inhibitory activity of Nanog-C or CD2 against Rad51 may implicate them widely into Rad51-related basic research and therapeutic application studies. The application potentials urged us to explore direct delivery of these Nanog fragments into cells. Synthetic nanosized materials and cell-penetrating peptide (CPP) are capable of facilitating direct uptake of peptide drugs into mammalian cells [72, 73]. Firstly, we utilized the zeolitic imidazolate framework-8 (ZIF-8) system, a well-established nano-scale porous material for drug delivery with exceptional thermal stability and chemical resistance [74, 75]. Bacterially purified Nanog fragments were encapsulated in the micropores of ZIF-8, forming nanosystems of C@ZIF-8, CD2@ZIF-8 and N@ZIF-8, respectively. Transmission electron microscopy (TEM) monitored their images. Results showed that ZIF-8 lost its typical rhombic dodecahedron shape and displayed round morphology suggesting successful peptide loading (Fig. 6a, the upper panel; [76]). Ultraviolet-visible spectroscopy (UV-Vis) analysis results confirmed successful loading of Nanog fragments in ZIF-8 (Fig. 6a, the lower panel; [76]). Next, we treated HCT116 cells with CPT for 6 h. Upon CPT removal, we added every Nanog fragment@ZIF-8 into fresh medium, respectively, to monitor its effect on γH2AX removal (Fig. 6b). Known Rad51 inhibitor B02 serves as a positive control [77]. Results of the western blotting assay showed that all the three Nanog fragments were successfully delivered into cells by ZIF-8 (Fig. 6c, d). Importantly, both C and CD2 released from ZIF-8 displayed strong inhibitory effect on γH2AX removal (Fig. 6c). Of note, both fragments displayed longer-lasting inhibition than B02 (Fig. 6c). Consistently, N@ZIF-8 failed in this effect (Fig. 6d). On the other hand, we employed the trans-activator of transcription (TAT)-mediated peptide delivery strategy [78, 79]. The 6xHis-tagged C and CD2 fused with the TAT fragment were bacterially expressed and purified, respectively, and directly added into the culture medium of HCT116 after 6-h CPT treatment. Western blotting assay showed successful uptake of Nanog fragments into cells. Of note, both fragments endocytosed exerted inhibitory effect on γH2AX removal (Fig. 6e).

## Discussion

H2AX is a highly-conserved histone H2A variant. In response to DSBs, it is phosphorylated to form γH2AX that activates DDR through recruiting other DDR-related factors to the lesion sites [80]. Unexpectedly, it is detected in unperturbed ES cells and early embryos, and quickly repressed upon cell differentiation [37, 41, 81]. Rapid reduction of Nanog by differentiation could release its inhibition of Rad51 to achieve successful removal of accumulated γH2Ax (Supplementary Fig. S7a–c). Similarly, DNA damage treatment also results in Nanog downregulation in ES cells [82] (Supplementary Fig. S7d), releasing Rad51 to exert repair of DNA damage. Of note, differentiation did not alter the total expression level of H2AX (Supplementary Fig. S7c). Interestingly, besides γH2AX activation, Nanog elevation simultaneously induces H3K14 acetylation to promote open chromatin formation in skin cancer cells [83]. It appears consistent with the pivotal role of Nanog in regulating the epigenetic landscape in ES cells [45, 84]. It is known that ES and ICM cells exhibit global open chromatin architecture that is required by stemness maintenance and rapid response to differentiation signals [85–88]. We speculate that γH2AX is consequence of Nanog-induced chromatin remodeling and possibly acts as one of the stemness-related chromatin properties. Interestingly, overexpression of either Nanog-C or CD2 led to induction of H3K14 acetylation and reduction of H3K27 methylation, suggesting open chromatin formation (Supplementary Fig. S7e). This observation indicates that Nanog-regulated chromatin remodeling is independent of its transcription regulatory activity. On the other hand, Rad51 foci are detected in unperturbed ES cells [41, 42]. The pre-existing Rad51 in chromatin might be poised for rapid γH2AX removal upon differentiation so as to achieve differentiated cell-favored condensed chromatin landscape efficiently. Alternatively, since open chromatins display higher susceptibility to DSBs than condensed chromatin [89, 90] pre-existing Rad51 foci might allow a rapid error-free repair once damages occur.

However, there are inconsistent observations for the roles of γH2AX in ES cells. Turinetto et al. showed that *H2AX* knockout impaired the self-renewal of mouse ES cells, which can be rescued by wild-type H2AX but not H2AX-S139A mutant [37], suggesting critical involvement of γH2AX in ES cell maintenance. In contrast, Andang et al. showed that *H2AX* depletion resulted in increased proliferation of mouse ES cells [91]. Intriguingly, a more recent study reported that reduction of γH2AX failed in altering proliferation of mouse ES cells [42]. Inconsistent observations were also obtained by a group of studies seeking to identify the kinases responsible for γH2AX activation in ES cells. Three phosphatidyl-inositol-3-kinase protein kinase family members have been well documented for their roles in catalyzing γH2AX formation, including ataxia telangiectasia mutated (ATM), DNA dependent protein kinase catalytic subunit (DNA-PKcs) and ATM and Rad3-related (ATR) [25, 92]. Since ATR inhibition results in massive death of ES cells, Turinetto et al. checked the effect of ATM or DNA-PKcs inhibition on γH2AX activation. They showed that inhibition of either kinase markedly reduced γH2AX but failed in completely abolished it [37], suggesting that ATM or DNA-PKcs at least partially contribute to the high level of γH2AX in mouse ES cells. However, Ahuja et al. did not observe γH2AX reduction after ATM inhibition, and they suggested ATR as the key kinase for γH2AX activation in ES cells [42]. To reconcile these paradoxical observations requires further studies to uncover the biological significance of γH2AX and mechanisms underlying γH2AX regulation in ES cells in future.

To develop inhibitors targeting key components of DNA damage repair signaling is an important avenue to improve the effectiveness of chemo- or radiotherapy for cancers [93]. Rad51 overexpression is widely detected in human cancers and closely associated with therapy resistance and poor prognosis [64–71]. This consequence follows from enhanced repair of genotoxic therapy-induced DNA damage by Rad51 elevation which prevents damage-caused cancer cell death. Thus, artful manipulation of Rad51 activity seems critical to improve the effectiveness of these therapies. Some Rad51 inhibitors have shown application potentials in cancer therapy. For instance, B02 ((E)-3-benzyl-2-(2-(pyridin-3-yl) vinyl) quinazolin-4(3H)-one), a specific small chemical inhibitor against Rad51, increases the killing effect of chemotherapeutic agent cisplatin on breast cancer cells [77]. Besides biological effect, concerns of toxicity are always raised for chemical inhibitor applications in human disease treatment. Nanog is an endogenous cellular protein with a short half-life [94], which determines its low cell toxicity and avoids of the risk in excessively inhibiting Rad51 activity since Rad51 is critical for genome integrity maintenance. However, Nanog promotes cancer development [46, 48, 49, 83]. Thus, direct use of Nanog appears not reliable to impede Rad51 elevation-reduced vulnerability of cancer cells to genotoxic therapies. Of note, the C or CD2 fragments maintain the ability of Rad51 inhibition while abandon the DB domain-dependent transcriptional activity of Nanog. Since Nanog promotes cancer cell growth and invasion mainly through its canonical transcriptional regulatory activity [47, 49, 95–98], use of these truncated fragments, rather than full-length Nanog, could realize improved effectiveness of genotoxic therapies and at the same time avoid of Nanog-promoted cancer development via transcriptional regulation. On the other hand, the capability of Nanog-C and CD2 in promoting open chromatin formation might act as an additional positive effect to enhance genotoxic therapy-induced cancer cell death since open chromatins display higher sensitivity to DNA damages than condensed counterparts (Supplementary Fig. S7e) [89, 90].

## Materials and methods

### Cell culture

Mouse E14 ES cells (ATCC) were cultured under a feeder-free condition at 37 °C with 5% CO_2_. The cells were maintained on gelatin-coated dishes in Dulbecco’s modified Eagle medium (DMEM; GIBCO), supplemented with 15% heat-inactivated fetal bovine serum (FBS; GIBCO), 0.1 mM β-mercaptoethanol (GIBCO), 2 mM L-glutamine, 0.1 mM MEM nonessential amino acid, 5000 units/ml penicillin/streptomycin and 1000 units/ml of LIF (ESGRO, ESG1107). HEK293, Hela and HCT116 cell lines were grown in DMEM (Hyclone) supplemented with 10% FBS (Invitrogen) at 37 °C with 5% CO2.

### Plasmids and cell transfection

Mouse Rad51, Nanog and Nanog fragments-expressing sequences were inserted into vector pcDNA4.0/TO(+) and peGFP-C1 respectively. Transfection was performed by using Lipofectamine 2000 (Invitrogen).

### Protein purification and Coomassie brilliant blue staining

The recombinant GST-tagged proteins were expressed in BL21, and then inducted with 0.2 mM IPTG at 16 °C and conjugated to Glutathione-Sepharose beads (GE Healthcare) in lysis buffer (50 mM Tris pH8.0, 10% glycerol, 0.3 M NaCl, 2 mM EDTA, 0.1% Triton X-100, 3 mM DTT, 1 mM PMSF, 1% protease inhibitor cocktail). After a 1.5 h incubation at 4 °C, the supernatant was removed with a 5 min spin at 1000 rpm, and the beads were washed three times with the lysis buffer at 4 °C. The recombinant GST-tagged proteins were eluted from the beads with the elution buffer (50 mM Tris pH 8.0, 10% glycerol, 0.3 M NaCl, 0.1% Triton X-100, 0.5 mM PMSF, 10 mM â-mercaptoethanol, 15 mM GSH).

The recombinant 6×His-tagged proteins were conjugated to Ni superflow beads in wash buffer (20 mM Hepes pH7.5, 10% glycerol, 1 M NaCl, 0.2% Triton X-100, 25 mM imidazole, 10 mM â-mercaptoethanol, 0.5 mM PMSF, 1% protease inhibitor cocktail). The beads were washed 5 times at 4 °C. Proteins were eluted with the buffer containing 20 mM Hepes pH7.5, 10% glycerol, 0.3 M NaCl, 0.35 M imidazole, 0.1% Triton X-100, 10 mM â-mercaptoethanol, 0.5 mM PMSF and 1% protease inhibitor cocktail.

Proteins were separated by SDS-PAGE gel and stained with Coomassie Brilliant Blue R-250 for 30 min. The gel was destained by the solution containing 25% methanol and 10% acetic acid at room temperature.

### Protein extraction and concentration measurement

Total protein was extracted by lysing cells with the whole cell extraction buffer (50 mM Tris; 150 mM NaCl; 1% NP40; 10% glycerol; 1 mM EDTA; 1 mM PMSF). Histones were obtained by the acid extraction assay. Cells were incubated in Triton extraction buffer with 0.5% Triton-X-100 and 2 mM PMSF for 10 min on ice. The suspension was centrifuged to collect pellets. The pellets obtained were re-suspended with 0.2 N HCl overnight. Concentration of Protein samples were determined by using the Coomassie blue (Bradford) Protein Assay. The standard and sample solutions were mixed with the Bradford reagent to measure their absorbance at 595 nm. The data of the standard solutions was plotted as the standard response curve, from which and the concentration of protein samples was calculated.

### Western blotting

Total protein (50 μg) or histone (5 μg) were separated by SDS-PAGE and transferred to PVDF membrane(GE Healthcare). The membrane was blocked with 5% milk and probed with specific primary antibodies and secondary antibodies. The blots were developed with ECL Advance Western Blotting Detection Kit (Amersham, #34080). The antibodies used in this study include Anti-Nanog antibody (Bethyl, A300-397A), anti-Rad51 antibody (Abcam, Ab63801), anti-HA antibody (Bethyl, A190-108A), anti-γH2AX antibody (Bethyl, A300-081A), anti-H3 antibody (Abcam, ab1791), anti-GST antibody (Santa Cruz, sc-138), anti-GFP (Abcam, ab290), anti-β-tubulin (Santa Cruz, sc-166729) and anti-GAPDH antibody (Bethyl, A300-641A).

### Co-immunoprecipitation

Protein samples were immunoprecipitated with antibody-conjugated Protein-G beads (GE Healthcare) and rotated at 4 °C overnight. The beads were washed for four times with 1 ml of cold NP40 lysis buffer containing protease inhibitors (Roche). The beads were then boiled for 10 min in the presence of 2× sample buffer and the eluted proteins were fractionated by SDS-PAGE. Proteins were detected by immunoblotting as described above.

### GST pull-down assay

Purified GST-tagged protein were precleared with Glutathione Sepharose 4B (GE Healthcare) for 1.5 h and incubated with His-tagged fusion proteins at 4 °C overnight. Protein-bound sepharose beads were washed 4 times with lysis buffer and eluted in SDS-PAGE sample buffer. Eluted proteins were analyzed by immunoblotting.

### Immunofluorescence staining assay

Cells were seeded on glass coverslips coated with poly-L-lysine (Solarbio). After 48 h, cells were washed with ice-cold PBS. Next, 4% paraformaldehyde (Sigma) in PBS was used to fix cells for 30 min at room temperature, after which the cells were permeabilized by 10-min treatment with 0.4% NP-40. The cells were blocked with PBS containing 3% bovine serum albumin (ANRESCO) for 30 min at room temperature, and subsequently incubated with anti-γH2AX antibody (Millipore, 05616) or rabbit anti-Rad51 antibody (Abcam, Ab63801) at 4 °C overnight. The cells were then washed by using PBS buffer, and then incubated with Alexa 488 secondary antibodies (Invitrogen) and DAPI (Sigma-Aldrich) for 30 min at room temperature in the dark. The cells were washed with PBS for 3 times. Immunofluorescence images were analysed by the Zeiss780 Confocal Microscope.

### RNA isolation, reverse transcription and quantitative real-time RT-PCR (qRT-PCR) analysis

Total RNAs were extracted using Trizol (Invitrogen, 15596018). cDNA synthesis was performed with 500 ng of total RNA using TransScript^®^ All-in-One First-Strand cDNA Synthesis SuperMix (TransGen, AT341-01) according to the manufacturer’s instructions. mRNA levels were measured by qRT-PCR analysis based on SYBR^®^ Premix Ex Taq™ (Takara, RR420A) with the BioRad real-time PCR machine. Results were normalized to β-actin. All the primers used in the study give rise to single product with the right size in agarose gel analysis. The data are presented as the mean ± SD (*t*-test; ***p < 0.001; **p < 0.01; *p < 0.05).

### Electrophoretic mobility shift assay

The single-stranded probes were labeled with biotin at the 5' termini. The sequence of the proble is 5'-AAATCAATCTAAAGTATATATGAGTAAACTTGGTCTGACAGTTACCAATGCTTAATCAGTGAGGCACCTATCTCAGCGATCTGTCTATTT-3'. 1 ng of probes were mixed with 1 μg of polydG/dC (Amersham), 2 μl of 5× reaction buffer (10 mM HEPES, pH7.5, 10 mM KCl, 10 mM MgCl2, 1 mM DTT, 1 mM EDTA, 10% glycerol), and 120 ng of purified proteins. The reaction mixtures were incubated at room temperature for 20 min. The different complexes in the reaction mixtures were separated by electrophoresis by using 10% DNA PAGE gels which had pre-run for 1 h at 4 °C. The gels were transferred to Biodyne B nylon membranes (Pierce Biotechnologies) and detected using the LightShift Chemiluminescent EMSA kit (Pierce Biotechnologies).

### Neutral comet assay

Harvested cells were resuspend in PBS buffer with the concentration of 2 × 10^5^ cells/ml. 1% normal melting point agarose was prepared on a frosted glass and solidified slowly at 4 °C. The cell suspension was mixed with low melting point agarose at the ratio of 1:10. Load 200 μl onto the Slides and spread them. After the agarose was solidified, slides were treated with the solution containing 2.5 M NaCl, 10 mM Tris-base, 100 mM EDTA, 1% TritonX-100 and 10% DMSO) for 1.5 h. Next, the slides were incubated with the unwinding buffer (0.05 M Tris-base,0.15 M Sodium Acetate) at 4 °C in the dark for 30 min, and then subjected to electrophoresis in neutral electrophoresis buffer (0.1 M Tris, 0.3 M sodium acetate, pH 8.5) at 4 °C (20 V for 45 min). The slides were then washed by 5-min incubation with the neutralization buffer (0.4 M Tris, PH7.5) at 4 °C for three times. Immerse slides in 70% ethanol for 30 min at room temperature. The slides were stained with SYBR^®^Gold (1:10000; Invitrogen) for 30 min. Tail moments and the percentage of tail DNA were measured by the CASP software.

### Synthesis of Nanog fragments@ZIF-8

Weigh 0.5 mmol of Zn(NO_3_)_2_·2H_2_O and 10 mmol of 2-Methylimidazole, respectively, and mix in 10 ml deionized water for 20 min by stirring. Put the mixture in a PTFE reactor and heat at 120 °C for 30 min. Allow the mixture to slowly cool down to room temperature. Centrifuge it at 7000 rpm and wash for 4 times using deionized water and 100% ethanol, respectively. Collect the pallet and dry it under vacuum to obtain ZIF-8. To obtain Nanog fragments@ZIF-8 suspension, mix 0.6 mg Nanog fragments with 30 mg ZIF-8 in 1.2 ml deionized water, and stir at 4 °C for 5 h. Centrifuge at 7000 rpm for 15 min to collect the pellet. Wash with deionized water for 3 times and resuspend in 1 ml PBS.

## Supplementary information

email reply from co-authors confirming that they agree to their positions in the author list.

Supplementary Figure Legends

Reproducibility checklist for all figures

Supplementary Figure S1

Supplementary Figure S2

Supplementary Figure S3

Supplementary Figure S4

Supplementary Figure S5

Supplementary Figure S6

Supplementary Figure S7

## Data Availability

All data generated or analyzed during this study are available from the corresponding author on reasonable request.
