# Supplementary material for: Identification of Nanog as a novel inhibitor of Rad51
Source: Cell Death Dis. 2022 Feb 26;13(2):193. doi: 10.1038/s41419-022-04644-9 (PMC8882189; doi:10.1038/s41419-022-04644-9)
Supplement: Supplementary file 1 — email reply from co-authors confirming that they agree to their positions in the author list. [file 41419_2022_4644_MOESM1_ESM.pdf]

Author 1#: Ying Xin:

Re:Rad51 manuscript

发件人：辛颖<18331093933@163.com>
时　间：2022年1月26日(星期三) 中午11:28
收件人：zhangww<zhangww@cnu.edu.cn>

Dear teacher,
I agree to my position in the author list.

Congratulations!
Ying Xin

在 2022-01-26 09:28:33，"张玮玮" <zhangww@cnu.edu.cn> 写道：

Dear authors,

Please reply to this email confirming that you agree to your position in the author list in our manuscript "Identification of Nanog as a novel inhibitor of Rad51" which has been provisionally accepted by the journal Cell Death & Disease.

The author list information shows in the following:

Ying Xin[1#], Juanjuan Wang[1#], Yahong Wu[1#], Qianqian Li[1], Mingyang Dong[1], Chang Liu[1], Qijia He[1], Ruifeng Wang[1], Dian Wang[1], Sen Jiang[2], Wei Xiao[1], Yang Tian[2*], Weiwei Zhang[1*]

1: College of Life Sciences, Capital Normal University, Beijing, China

2: Department of Chemistry, Capital Normal University, Beijing, China

# *These authors contributed equally to this work.*

* *Corresponding author*

Thanks and regards,

Weiwei Zhang

Author 2#: Juanjuan Wang: wjj521218@163.com

Re:Rad51 manuscript

发件人：王娟娟<wjj521218@163.com>
时 间：2022年1月26日(星期三) 中午11:30
收件人：zhangww<zhangww@cnu.edu.cn>

Dear Miss Zhang,

I agree to my position in the author list.

Juanjuan Wang

Thank you very much.

Best regards,
Juanjuan

--
首都师范大学 实验室732
北京市西三环北路105号，100048
电话：+86 15120086286

College of Life Sciences,Capital Normal University
#105, Xi San Huan Bei Road, Hai Dian District, Beijing 100048, China
TEL:+86 15120086286

在 2022-01-26 09:28:33，"张玮玮" <zhangww@cnu.edu.cn> 写道：

> Dear authors,
>
> Please reply to this email confirming that you agree to your position in the author list in our manuscript "Identification of Nanog as a novel inhibitor of Rad51" which has been provisionally accepted by the journal Cell Death & Disease.
>
> The author list information shows in the following:
>
> Ying Xin[1#], Juanjuan Wang[1#], Yahong Wu[1#], Qianqian Li[1], Mingyang Dong[1], Chang Liu[1], Qijia He[1], Ruifeng Wang[1], Dian Wang[1], Sen Jiang[2], Wei Xiao[1], Yang Tian[2*], Weiwei Zhang[1*]
>
> *1: College of Life Sciences, Capital Normal University, Beijing, China*
>
> *2: Department of Chemistry, Capital Normal University, Beijing, China*

Author 3#: Yahong Wu: 17835061377@163.com

回复: Rad51 manuscript

发件人：17835061377@163.com<17835061377@163.com>
时　间：2022年1月26日(星期三) 上午10:38
收件人：zhangww<zhangww@cnu.edu.cn>

I agree to my position in the author list.

Yahong Wu

发自vivo智能手机

Dear authors,

Please reply to this email confirming that you agree to your position in the author list in our manuscript "Identification of Nanog as a novel inhibitor of Rad51" which has been provisionally accepted by the journal Cell Death & Disease.

The author list information shows in the following:

Ying Xin[1#], Juanjuan Wang[1#], Yahong Wu[1#], Qianqian Li[1], Mingyang Dong[1], Chang Liu[1], Qijia He[1], Ruifeng Wang[1], Dian Wang[1], Sen Jiang[2], Wei Xiao[1], Yang Tian[2*], Weiwei Zhang[1*]

1: College of Life Sciences, Capital Normal University, Beijing, China

2: Department of Chemistry, Capital Normal University, Beijing, China

[#] *These authors contributed equally to this work.*

* *Corresponding author*

Thanks and regards,

Weiwei Zhang

张玮玮
首都师范大学/生命科学学院
北京市海淀区西三环北路105号

Author 4#: Qianqian Li: 2200802102@cnu.edu.cn

回复：Rad51 manuscript

发件人：李倩倩<2200802102@cnu.edu.cn>
时　间：2022年1月26日(星期三) 上午10:41
收件人：zhangww<zhangww@cnu.edu.cn>

Dear:
I agree to my position in the author list in our manuscript "Identification of Nanog as a novel inhibitor of Rad51" which has been provisionally accepted by the journal Cell Death & Disease.

Qianqian Li

---------------原始邮件---------------

发件人: 张玮玮
发送时间: 2022年01月26日 09:28
收件人: 18331093933<18331093933@163.com>，wjj521218，17835061377<17835061377@163.com>，2200802102<2200802102@cnu.edu.cn>，dmydyxy，radientliu，342767884<342767884@qq.com>，ruifengwxk，857405710<857405710@qq.com>，2376332867<2376332867@qq.com>，weixiao，tianyang，萧伟
主题: Rad51 manuscript

Dear authors,

Please reply to this email confirming that you agree to your position in the author list in our manuscript "Identification of Nanog as a novel inhibitor of Rad51" which has been provisionally accepted by the journal Cell Death & Disease.

The author list information shows in the following:

Ying Xin[1#], Juanjuan Wang[1#], Yahong Wu[1#], Qianqian Li[1], Mingyang Dong[1], Chang Liu[1], Qijia He[1], Ruifeng Wang[1], Dian Wang[1], Sen Jiang[2], Wei Xiao[1], Yang Tian[2*], Weiwei Zhang[1*]

1: College of Life Sciences, Capital Normal University, Beijing, China

2: Department of Chemistry, Capital Normal University, Beijing, China

# *These authors contributed equally to this work.*

** Corresponding author*

Thanks and regards,

Weiwei Zhang

张玮玮
首都师范大学/生命科学学院
北京市海淀区西三环北路105号

Author 5#: Mingyang Dong: dmydyxy@163.com

Re:Rad51 manuscript

发件人：董明阳<dmydyxy@163.com>
时　间：2022年1月26日(星期三) 中午12:56
收件人：zhangww<zhangww@cnu.edu.cn>

Dear Professor Zhang,

I agree with the position in the author list of our manuscript "Identification of Nanog as a novel inhibitor of Rad51"

Thanks and regards,

Mingyang Dong

在 2022-01-26 09:28:33，"张玮玮" <zhangww@cnu.edu.cn> 写道：

> Dear authors,
>
> Please reply to this email confirming that you agree to your position in the author list in our manuscript "Identification of Nanog as a novel inhibitor of Rad51" which has been provisionally accepted by the journal Cell Death & Disease.
>
> The author list information shows in the following:
>
> Ying Xin[1#], Juanjuan Wang[1#], Yahong Wu[1#], Qianqian Li[1], Mingyang Dong[1], Chang Liu[1], Qijia He[1], Ruifeng Wang[1], Dian Wang[1], Sen Jiang[2], Wei Xiao[1], Yang Tian[2*], Weiwei Zhang[1*]
>
> *1: College of Life Sciences, Capital Normal University, Beijing, China*
>
> *2: Department of Chemistry, Capital Normal University, Beijing, China*
>
> [#] *These authors contributed equally to this work.*
>
> * *Corresponding author*
>
> Thanks and regards,
>
> Weiwei Zhang

Author 6#: Chang Liu: radientliu@163.com

Re:Rad51 manuscript

发件人：radientliu<radientliu@163.com>
时　间：2022年1月26日(星期三) 中午11:47
收件人：zhangww<zhangww@cnu.edu.cn>

Dear Professor Zhang,

I agree with the position in the author list of our manuscript "Identification of Nanog as a novel inhibitor of Rad51"

Thanks and regards,

Chang Liu

在 2022-01-26 09:28:33，"张玮玮" <zhangww@cnu.edu.cn> 写道：

> Dear authors,
>
> Please reply to this email confirming that you agree to your position in the author list in our manuscript "Identification of Nanog as a novel inhibitor of Rad51" which has been provisionally accepted by the journal Cell Death & Disease.
>
> The author list information shows in the following:
>
> Ying Xin[1#], Juanjuan Wang[1#], Yahong Wu[1#], Qianqian Li[1], Mingyang Dong[1], Chang Liu[1], Qijia He[1], Ruifeng Wang[1], Dian Wang[1], Sen Jiang[2], Wei Xiao[1], Yang Tian[2*], Weiwei Zhang[1*]
>
> *1: College of Life Sciences, Capital Normal University, Beijing, China*
>
> *2: Department of Chemistry, Capital Normal University, Beijing, China*
>
> [#] *These authors contributed equally to this work.*
>
> * *Corresponding author*
>
> Thanks and regards,
>
> Weiwei Zhang
>
> **张玮玮**
> 首都师范大学/生命科学学院

Author 7#: Qijia He: 342767884@qq.com

回复：Rad51 manuscript

发件人：hqj<342767884@qq.com>
时　间：2022年1月26日(星期三) 上午10:12
收件人：zhangww<zhangww@cnu.edu.cn>

I agree to my position in the author list.

Qijia He

------------------ 原始邮件 ------------------

发件人: "张玮玮" <zhangww@cnu.edu.cn>;
发送时间: 2022年1月26日(星期三) 上午9:28
收件人: "18331093933"<18331093933@163.com>;"wjj521218"<wjj521218@163.com>;"17835061377"<17835061377@163.com>;"2200802102"<2200802102@cnu.edu.cn>;"dmydyxy"<dmydyxy@163.com>;"radientliu"<radientliu@163.com>;"HQJ"<342767884@qq.com>;"ruifengwxk"<ruifengwxk@gmail.com>;"857405710"<857405710@qq.com>;"2376332867"<2376332867@qq.com>;"weixiao"<weixiao@cnu.edu.cn>;"tianyang"<tianyang@cnu.edu.cn>;"萧伟"<wei.xiao@usask.ca>;
主题: Rad51 manuscript

Dear authors,

Please reply to this email confirming that you agree to your position in the author list in our manuscript "Identification of Nanog as a novel inhibitor of Rad51" which has been provisionally accepted by the journal Cell Death & Disease.

The author list information shows in the following:

Ying Xin[1#], Juanjuan Wang[1#], Yahong Wu[1#], Qianqian Li[1], Mingyang Dong[1], Chang Liu[1], Qijia He[1], Ruifeng Wang[1], Dian Wang[1], Sen Jiang[2], Wei Xiao[1], Yang Tian[2*], Weiwei Zhang[1*]

1: College of Life Sciences, Capital Normal University, Beijing, China

2: Department of Chemistry, Capital Normal University, Beijing, China

# These authors contributed equally to this work.

** Corresponding author*

Thanks and regards,

Weiwei Zhang

张玮玮
首都师范大学/生命科学学院
北京市海淀区西三环北路105号

Author 8#: Ruifeng Wang: ruifengwxk@gmail.com

Re: Rad51 manuscript

发件人：ruifengwxk<ruifengwxk@gmail.com>
时　间：2022年1月26日(星期三) 下午3:51
收件人：zhangww<zhangww@cnu.edu.cn>

Dear Prof. Zhang,
Yes, I agree. Thanks!

Best regards,
Reifeng

发自我的iPad

在 26.01.2022，02:28，张玮玮 <zhangww@cnu.edu.cn> 写道：

Dear authors,

Please reply to this email confirming that you agree to your position in the author list in our manuscript "Identification of Nanog as a novel inhibitor of Rad51" which has been provisionally accepted by the journal Cell Death & Disease.

The author list information shows in the following:

Ying Xin[1#], Juanjuan Wang[1#], Yahong Wu[1#], Qianqian Li[1], Mingyang Dong[1], Chang Liu[1], Qijia He[1], Ruifeng Wang[1], Dian Wang[1], Sen Jiang[2], Wei Xiao[1], Yang Tian[2*], Weiwei Zhang[1*]

1: College of Life Sciences, Capital Normal University, Beijing, China

2: Department of Chemistry, Capital Normal University, Beijing, China

# *These authors contributed equally to this work.*

* *Corresponding author*

Thanks and regards,

Weiwei Zhang

张玮玮
首都师范大学/生命科学学院
北京市海淀区西三环北路105号

Author 9#: Dian Wang: 857405710@qq.com

回复：Rad51 manuscript

发件人：王典<857405710@qq.com>
时　间：2022年1月26日(星期三) 上午10:08
收件人：zhangww<zhangww@cnu.edu.cn>

Dear professor,

I agree with my position in the author list.

Thanks,

Dian Wang

------------------ 原始邮件 ------------------

发件人: "张玮玮" <zhangww@cnu.edu.cn>;
发送时间: 2022年1月26日(星期三) 上午9:28
收件人: "18331093933"<18331093933@163.com>;"wjj521218"<wjj521218@163.com>;"17835061377"<17835061377@163.com>;"2200802102"<2200802102@cnu.edu.cn>;"dmydyxy"<dmydyxy@163.com>;"radientliu"<radientliu@163.com>;"342767884"<342767884@qq.com>;"ruifengwxk"<ruifengwxk@gmail.com>;"王典"<857405710@qq.com>;"2376332867"<2376332867@qq.com>;"weixiao"<weixiao@cnu.edu.cn>;"tianyang"<tianyang@cnu.edu.cn>;"萧伟"<wei.xiao@usask.ca>;
主题: Rad51 manuscript

Dear authors,

Please reply to this email confirming that you agree to your position in the author list in our manuscript "Identification of Nanog as a novel inhibitor of Rad51" which has been provisionally accepted by the journal Cell Death & Disease.

The author list information shows in the following:

Ying Xin[1#], Juanjuan Wang[1#], Yahong Wu[1#], Qianqian Li[1], Mingyang Dong[1], Chang Liu[1], Qijia He[1], Ruifeng Wang[1], Dian Wang[1], Sen Jiang[2], Wei Xiao[1], Yang Tian[2*], Weiwei Zhang[1*]

1: College of Life Sciences, Capital Normal University, Beijing, China

2: Department of Chemistry, Capital Normal University, Beijing, China

[#] These authors contributed equally to this work.

** Corresponding author*

Thanks and regards,

Weiwei Zhang

张玮玮
首都师范大学/生命科学学院

Author 10#: Sen Jiang: 2376332867@qq.com

回复：Rad51 manuscript

发件人：2376332867<2376332867@qq.com>
时　间：2022年1月26日(星期三) 上午9:45
收件人：zhangww<zhangww@cnu.edu.cn>

Dear Mrs Zhang,
I agree to my position in the author list.
Best Regards
Jiang sen

------------------ 原始邮件 ------------------

发件人: "张玮玮" <zhangww@cnu.edu.cn>;
发送时间: 2022年1月26日(星期三) 上午9:28
收件人: "18331093933"<18331093933@163.com>;"wjj521218"<wjj521218@163.com>;"17835061377"<17835061377@163.com>;"2200802102"<2200802102@cnu.edu.cn>;"dmydyxy"<dmydyxy@163.com>;"radientliu"<radientliu@163.com>;"342767884"<342767884@qq.com>;"ruifengwxk"<ruifengwxk@gmail.com>;"857405710"<857405710@qq.com>;"笨小孩"<2376332867@qq.com>;"weixiao"<weixiao@cnu.edu.cn>;"tianyang"<tianyang@cnu.edu.cn>;"萧伟"<wei.xiao@usask.ca>;
主题: Rad51 manuscript

Dear authors,

Please reply to this email confirming that you agree to your position in the author list in our manuscript "Identification of Nanog as a novel inhibitor of Rad51" which has been provisionally accepted by the journal Cell Death & Disease.

The author list information shows in the following:

Ying Xin[1#], Juanjuan Wang[1#], Yahong Wu[1#], Qianqian Li[1], Mingyang Dong[1], Chang Liu[1], Qijia He[1], Ruifeng Wang[1], Dian Wang[1], Sen Jiang[2], Wei Xiao[1], Yang Tian[2*], Weiwei Zhang[1*]

1: College of Life Sciences, Capital Normal University, Beijing, China

2: Department of Chemistry, Capital Normal University, Beijing, China

[#] *These authors contributed equally to this work.*

* *Corresponding author*

Thanks and regards,

Weiwei Zhang

张玮玮
首都师范大学/生命科学学院
北京市海淀区西三环北路105号

Author 11#: Wei Xiao：weixiao@cnu.edu.cn

Re: Rad51 manuscript

发件人：萧伟<wei.xiao@usask.ca>
时　间：2022年1月26日(星期三) 中午1:03
收件人：zhangww<zhangww@cnu.edu.cn>

I agree to my position as shown in the authorship list.

Wei Xiao

From: 张玮玮 <zhangww@cnu.edu.cn>
Date: Tuesday, January 25, 2022 at 7:28 PM
To: 18331093933 <18331093933@163.com>, wjj521218 <wjj521218@163.com>, 17835061377 <17835061377@163.com>, 2200802102 <2200802102@cnu.edu.cn>, dmydyxy <dmydyxy@163.com>, radientliu <radientliu@163.com>, 342767884 <342767884@qq.com>, ruifengwxk <ruifengwxk@gmail.com>, 857405710 <857405710@qq.com>, 2376332867 <2376332867@qq.com>, weixiao <weixiao@cnu.edu.cn>, tianyang <tianyang@cnu.edu.cn>, "Xiao, Wei" <wei.xiao@usask.ca>
Subject: Rad51 manuscript

CAUTION: External to USask. Verify sender and use caution with links and attachments. Forward suspicious emails to phishing@usask.ca

Dear authors,

Please reply to this email confirming that you agree to your position in the author list in our manuscript "Identification of Nanog as a novel inhibitor of Rad51" which has been provisionally accepted by the journal Cell Death & Disease.

The author list information shows in the following:

Ying Xin[1#], Juanjuan Wang[1#], Yahong Wu[1#], Qianqian Li[1], Mingyang Dong[1], Chang Liu[1], Qijia He[1], Ruifeng Wang[1], Dian Wang[1], Sen Jiang[2], Wei Xiao[1], Yang Tian[2*], Weiwei Zhang[1*]

1: College of Life Sciences, Capital Normal University, Beijing, China

2: Department of Chemistry, Capital Normal University, Beijing, China

# *These authors contributed equally to this work.*

* *Corresponding author*

Thanks and regards,

Author 12#: Yang Tian: tianyang@cnu.edu.cn

Re:Rad51 manuscript

发件人：田洋<tianyang@cnu.edu.cn>
时　间：2022年1月26日(星期三) 上午10:16
收件人：zhangww<zhangww@cnu.edu.cn>

Dear prof. Weiwei Zhang,

I am agreemnt with the position in the author lisht. Thank you for your kind email.

Best wishes,

Yang Tian

Department of Chemistry
Capital Normal University

田洋 联系我
首都师范大学

------------------ Original ------------------

From: "张玮玮"<zhangww@cnu.edu.cn>;
Date: Wed, Jan 26, 2022 09:28 AM
To: "18331093933"<18331093933@163.com>; "wjj521218"<wjj521218@163.com>; "17835061377"<17835061377@163.com>; "2200802102"<2200802102@cnu.edu.cn>; "dmydyxy"<dmydyxy@163.com>; "radientliu"<radientliu@163.com>; "342767884"<342767884@qq.com>; "ruifengwxk"<ruifengwxk@gmail.com>; "857405710"<857405710@qq.com>; "2376332867"<2376332867@qq.com>; "weixiao"<weixiao@cnu.edu.cn>; "tianyang"<tianyang@cnu.edu.cn>; "萧伟"<wei.xiao@usask.ca>;
Subject: Rad51 manuscript

Dear authors,

Please reply to this email confirming that you agree to your position in the author list in our manuscript "Identification of Nanog as a novel inhibitor of Rad51" which has been provisionally accepted by the journal Cell Death & Disease.

The author list information shows in the following:

Ying Xin[1#], Juanjuan Wang[1#], Yahong Wu[1#], Qianqian Li[1], Mingyang Dong[1], Chang Liu[1], Qijia He[1], Ruifeng Wang[1], Dian Wang[1], Sen Jiang[2], Wei Xiao[1], Yang Tian[2*], Weiwei Zhang[1*]

1: College of Life Sciences, Capital Normal University, Beijing, China

2: Department of Chemistry, Capital Normal University, Beijing, China

# *These authors contributed equally to this work.*

* *Corresponding author*

Thanks and regards,

Weiwei Zhang
