# Supplementary material for: Identification of Nanog as a novel inhibitor of Rad51
Source: Cell Death Dis. 2022 Feb 26;13(2):193. doi: 10.1038/s41419-022-04644-9 (PMC8882189; doi:10.1038/s41419-022-04644-9)
Supplement: Supplementary file 2 — Supplementary Figure Legends [file 41419_2022_4644_MOESM2_ESM.doc]

**[Supplementary Figure](https://www.nature.com/articles/sigtrans20169#MOESM16) Legends**

[Supplementary Fig. S](https://www.nature.com/articles/sigtrans20169#MOESM16)1 Oct4 and Sox2 fail in activating γH2AX. a, b Oct4 and Sox2 did not activate γH2AX. Mouse ES cells were transfected with the plasmid expressing HA-tagged Oct4 (a) or Sox2 (b). Transfection with empty vector was used as control. 48 hours after transfection, whole cell proteins and histones were extracted and subjected to SDS-PAGE, respectively. c Co-IP assay was used to detect the association of Rad51 with Oct4 and Sox2. Mouse ES cell extracts were subjected to co-IP with the antibody against Rad51, followed by western blotting with the antibodies against Oct4 and Sox2, respectively. 5% of whole cell extract for IP was used as the input control.

The data are based on three independent repeats.

[Supplementary Fig. S](https://www.nature.com/articles/sigtrans20169#MOESM16)2 Nanog prevents repairs of DSB in human cancer cells. a Representative cell images of the comet assay in HeLa cells. HeLa cells overexpressing Nanog were treated with CPT for 6 hours. The medium was changed to fresh CPT-free medium and allowed cells to grow for additional 4 and 8 hours, respectively. The cells transfected with the mock vector were used as controls. b The average percentages of DNA in tails were calculated.

The data are based on three independent repeats.

[Supplementary Fig. S](https://www.nature.com/articles/sigtrans20169#MOESM16)3 Nanog inhibits DSB repair through its C and CD2 regions. a Nanog-C specifically induced γH2AX activation. HEK293 cells were transfected with different amounts of plasmid expressing GFP-tagged Nanog-C (+: 2μg; ++: 4μg; +++: 6μg; ++++: 8μg). Whole cell proteins and histones were extracted and subjected to SDS-PAGE, respectively. The blot band intensities were quantitated by MultiGauge software ([Fujifilm](https://fujifilm.jp/business/lifescience/simaging/software/multigauge/downloade/input.cgi)). The data was normalized to loading controls with the antibody against H3 (The right panel). b, c Similar experiments with a were performed to examine the capability of the CD2 (b) and N (c) in activating γH2AX. d Representative cell images of the comet assay. 293 cells overexpressing Nanog-C or CD2 were treated with 2μM CPT for 4 and 8 hours, respectively. The medium was changed to fresh CPT-free medium and allowed cells to grow for additional 16 hours. The cells transfected with the mock vector were used as controls. e Tail moments of the samples from d were analyzed.

The data are based on three independent repeats, and presented as mean ± SEM. ***p < 0.001; **p < 0.01; *p < 0.05 (Student's t-test).

[Supplementary Fig. S](https://www.nature.com/articles/sigtrans20169#MOESM16)4 Nanog-C/CD2 overexpression did not result in change in cell cycle progression. The constructs expressing full-length, C and CD2 fragment of Nanog, were transfected into HeLa cells, respectively. After 48 hours, the cells were harvested for the FACS analysis (a). b Western blotting assay showing the expression levels of Nanog fragments.

The data are based on three independent repeats, and presented as mean ± SEM. **p < 0.01 (Student's t-test).

[Supplementary Fig. S](https://www.nature.com/articles/sigtrans20169#MOESM16)5 Nanog repressed Rad51 foci formation. a Nanog-overexpressed HeLa cells were stained with the anti-Rad51 antibody (red) and DAPI (blue). b Rad51 foci in the cells from a were counted and analysed. The nuclei containing more than 10 foci were considered to be positive. (c) and (d) Overexpression of Nanog fragments did not alter the expression level of Rad51. The constructs expressing full-length, C and CD2 of Nanog were transfected into HCT116 cells, respectively. The resulting cells 48 hours after transfection were analyzed by qRT-PCR (c) and western blotting assay (d).

The data are based on three independent repeats, and presented as mean ± SEM. **p < 0.01 (Student's t-test).

[Supplementary Fig. S](https://www.nature.com/articles/sigtrans20169#MOESM16)6 Nanog colocalizes at the lesion sites of DNA damage. a Nanog overexpression induced 53BP1 foci. The foci of 53BP1 were detected by the ICC staining assay. b Purified GST-tagged Nanog were conjugated to Glutathione-sepharose beads to capture 53BP1 with the whole cell lysis of HeLa cells. c, d Pearson correlation coefficient analysis of the foci correlation of Nanog with Rad51 (c) and 53BP1 (d). The ICC images were captured by the Opera Phenix High Content Screening System (PerkinElmer) and analyzed with the Harmony High Content Imaging and Analysis Software (PerkinElmer). Pearson correlation coefficient was calculated with Coloc2 plugin (https://imagej.net/plugins/coloc-2) in Fiji image. The data were visualized by the Custom made R scripts ([https://www.R-project.org/](https://www.r-project.org/)) and GraphPad 8.0.2.

The data are based on three independent repeats, and presented as mean ± SEM. ***p < 0.0001 (Student's t-test).

[Supplementary Fig. S](https://www.nature.com/articles/sigtrans20169#MOESM16)7 γH2AX activation is closely co-related with the undifferentiated state of mouse ES cells. a RA induced ES cells to differentiate. 48 hours after RA treatment, immunocytochemistry staining assay was performed to analyze the expression of Nanog (red) and Oct4 (green) in mouse ES cells without pre-extracting cellular soluble fractions. b γH2AX was reduced upon RA-induced ES cell differentiation. The mouse MEF cells under CPT treatment were used as controls. c γH2AX was reduced upon DMSO-induced ES cell differentiation. 1% DMSO treated mouse ES cells for 5 days. Whole cell extracts were obtained for western blotting analysis. d The effect of DNA damage on the expression of Nanog and Rad51. ES cells and HCT116 cells were treated with CPT for 6 hours, respectively. Whole cell proteins were extracted and subjected to SDS-PAGE with antibodies against Nanog and Rad51. e Nanog-C and CD2 promoted open chromatin formation. Histone extraction and whole cell proteins were obtained from HCT116 cells overexpressing GFP-tagged C or CD2 and subjected to SDS-PAGE. The cells transfected with the mock vector were used as controls.

The data are based on three independent repeats, and presented as mean ± SEM. **p < 0.01; *p < 0.05 (Student's t-test).
