# Supplementary figures and images for: Identification of Nanog as a novel inhibitor of Rad51
Source: Cell Death Dis. 2022 Feb 26;13(2):193. doi: 10.1038/s41419-022-04644-9 (PMC8882189; doi:10.1038/s41419-022-04644-9)

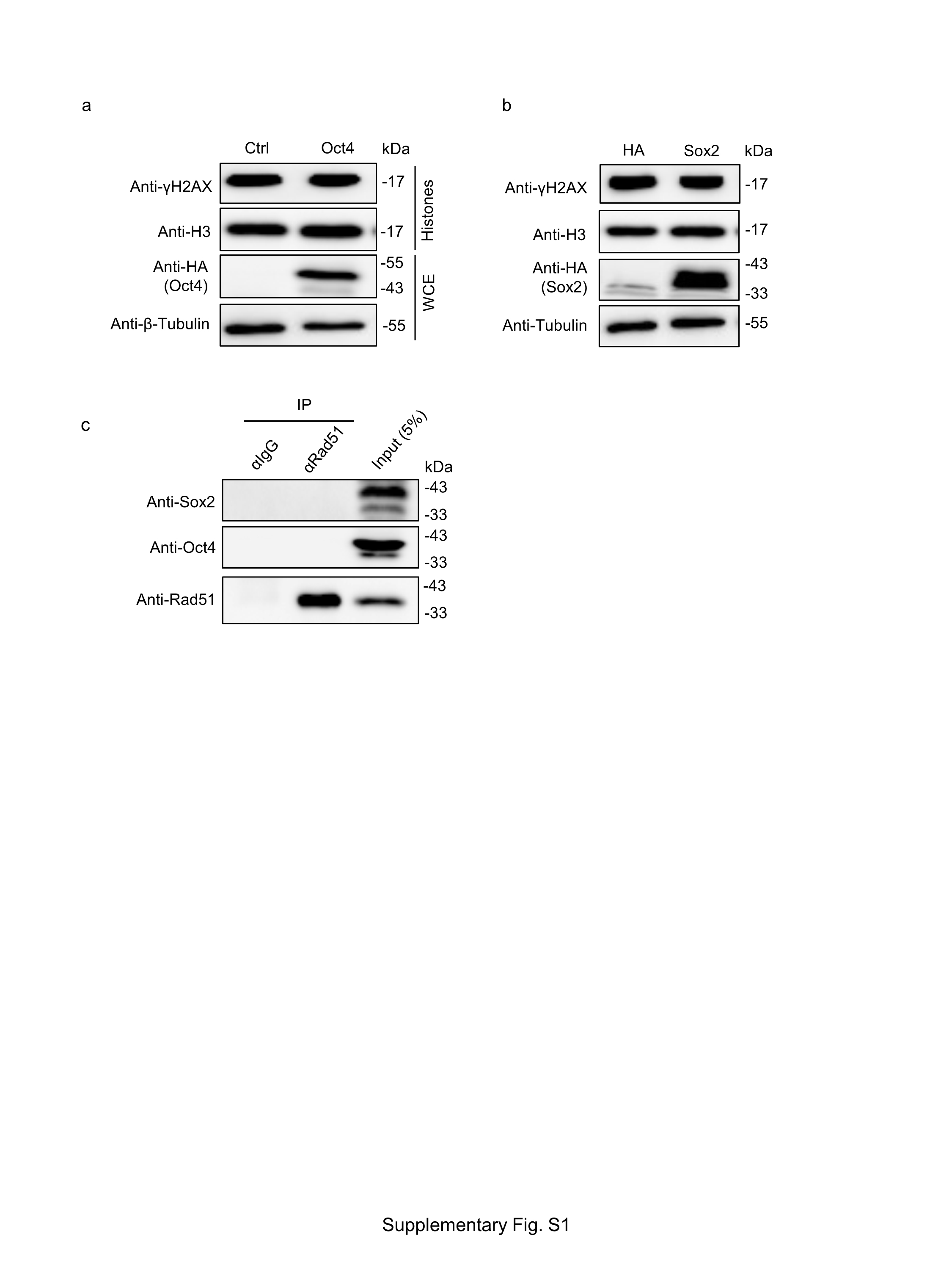

Supplement: Supplementary file 4 — Supplementary Figure S1 [file 41419_2022_4644_MOESM4_ESM.jpg]

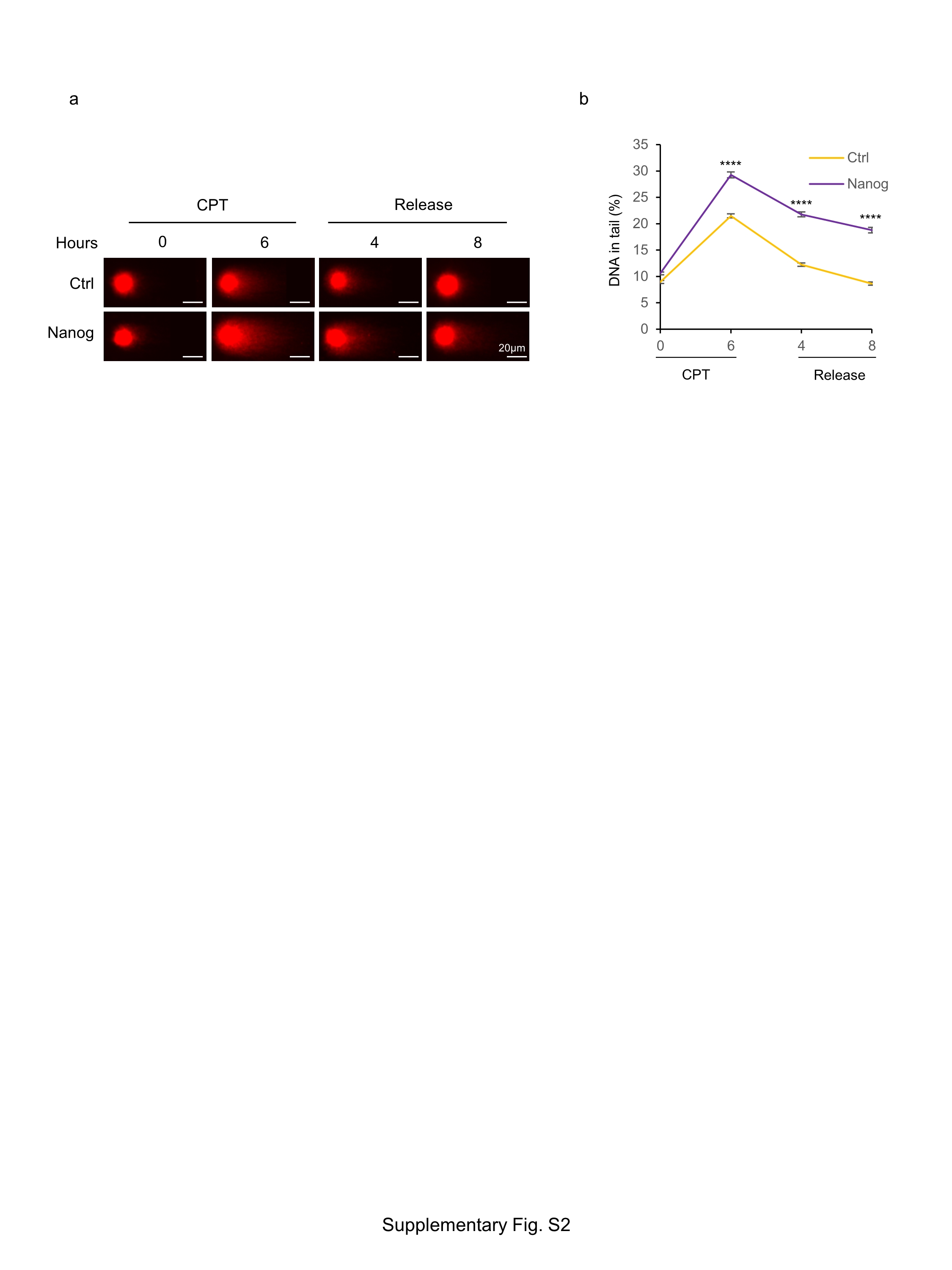

Supplement: Supplementary file 5 — Supplementary Figure S2 [file 41419_2022_4644_MOESM5_ESM.jpg]

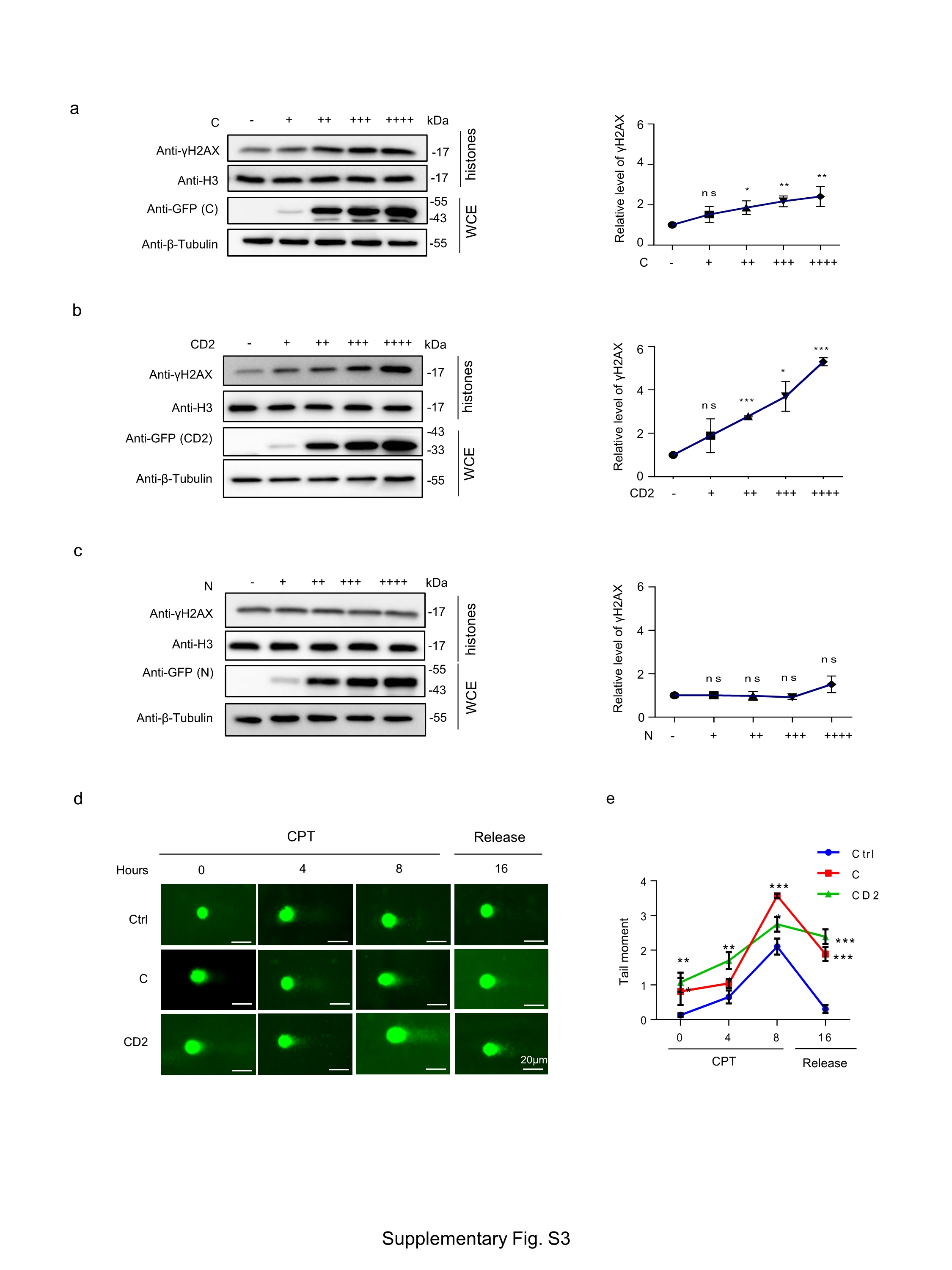

Supplement: Supplementary file 6 — Supplementary Figure S3 [file 41419_2022_4644_MOESM6_ESM.jpg]

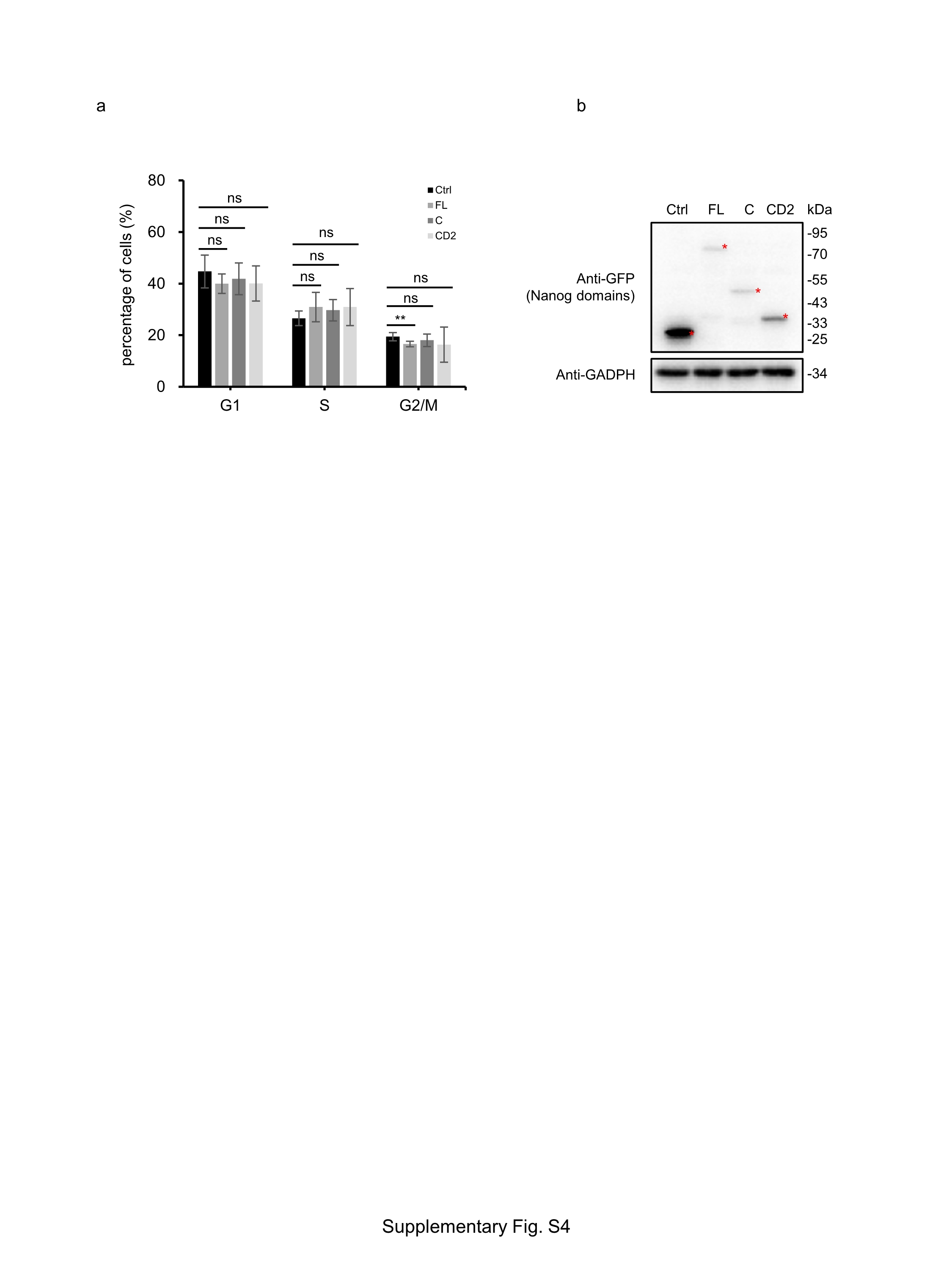

Supplement: Supplementary file 7 — Supplementary Figure S4 [file 41419_2022_4644_MOESM7_ESM.jpg]

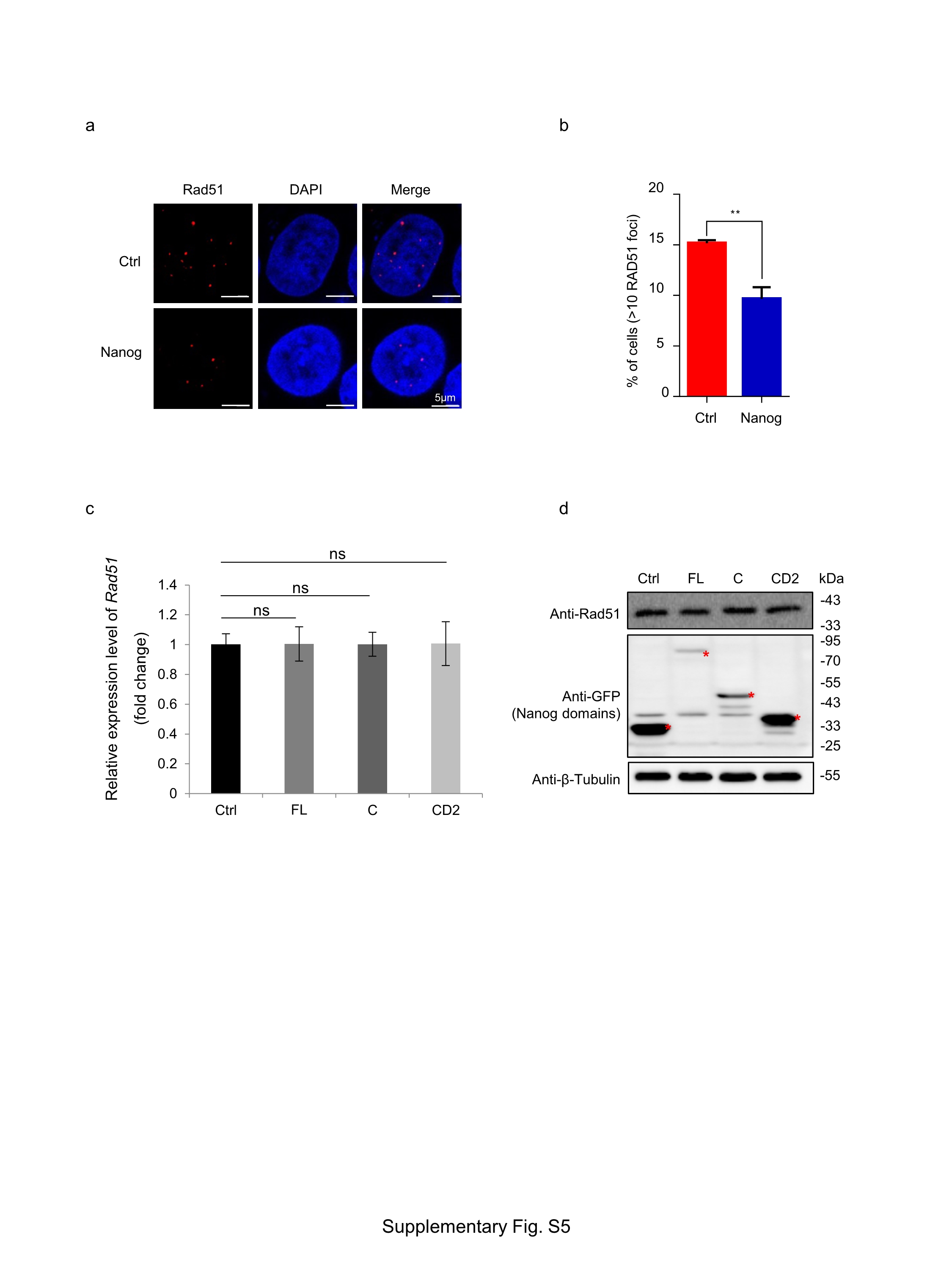

Supplement: Supplementary file 8 — Supplementary Figure S5 [file 41419_2022_4644_MOESM8_ESM.jpg]

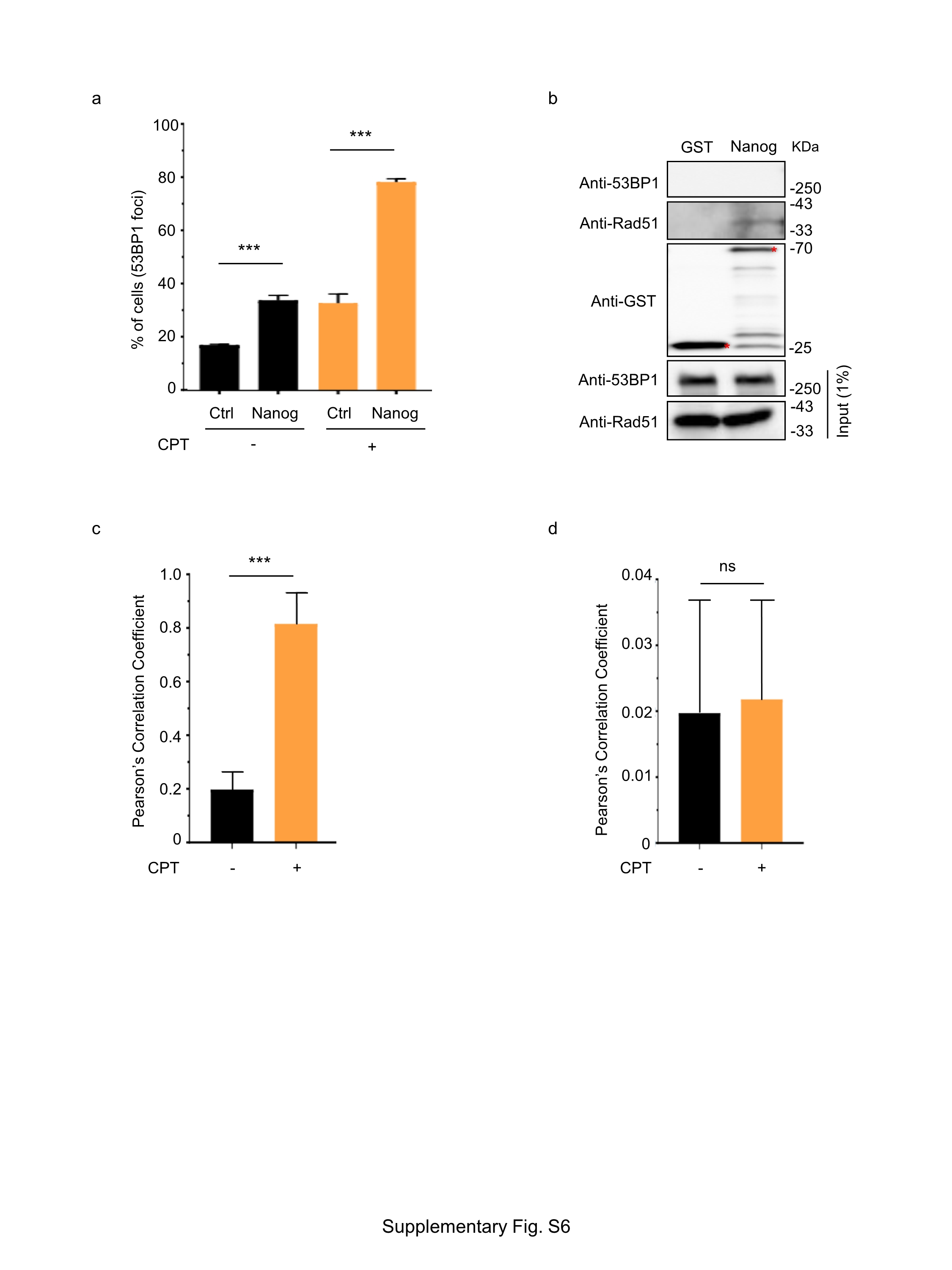

Supplement: Supplementary file 9 — Supplementary Figure S6 [file 41419_2022_4644_MOESM9_ESM.jpg]

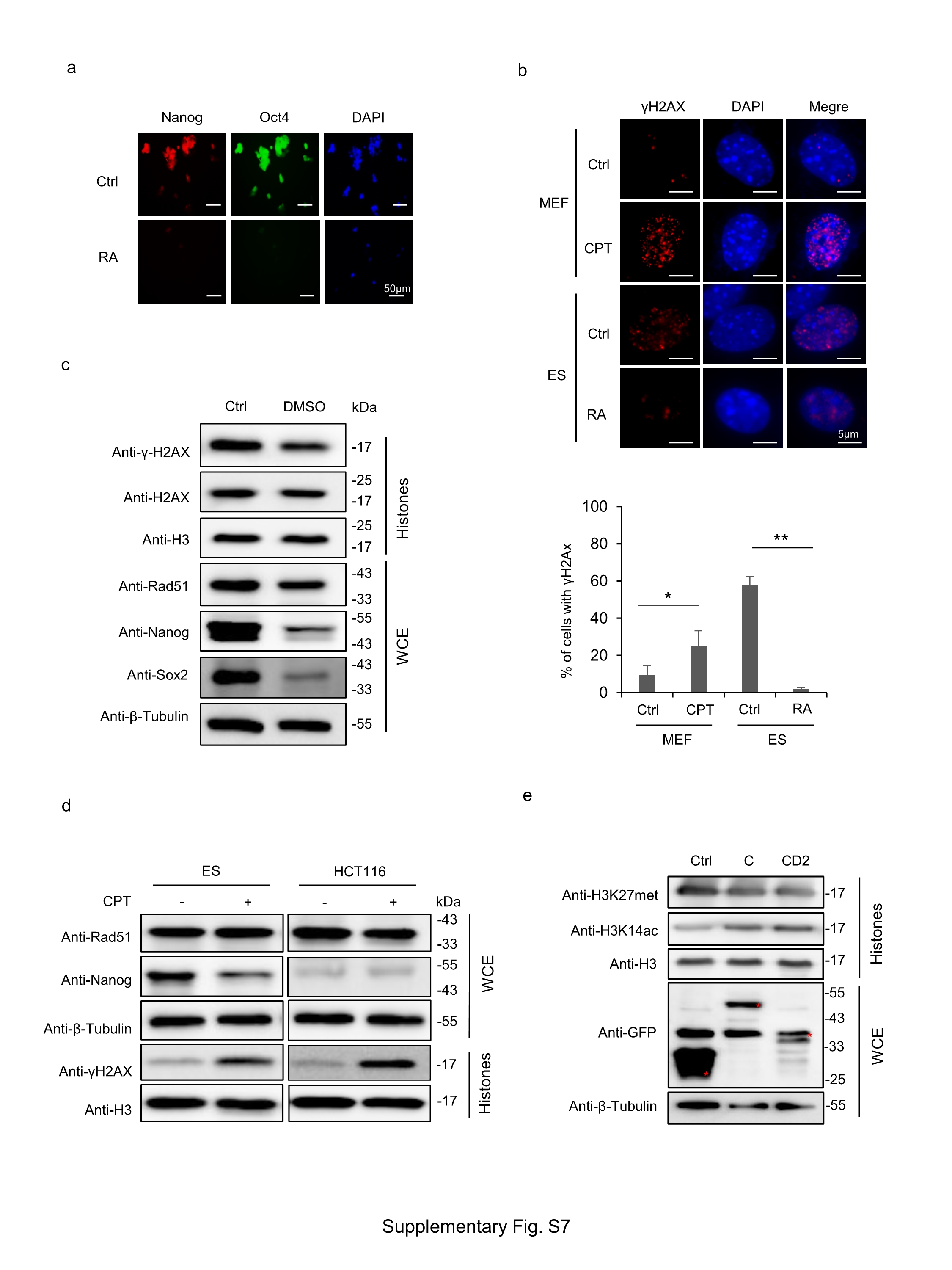

Supplement: Supplementary file 10 — Supplementary Figure S7 [file 41419_2022_4644_MOESM10_ESM.jpg]
